# Spin–phonon couplings in transition metal complexes with slow magnetic relaxation

**DOI:** 10.1038/s41467-018-04896-0

**Published:** 2018-07-03

**Authors:** Duncan H. Moseley, Shelby E. Stavretis, Komalavalli Thirunavukkuarasu, Mykhaylo Ozerov, Yongqiang Cheng, Luke L. Daemen, Jonathan Ludwig, Zhengguang Lu, Dmitry Smirnov, Craig M. Brown, Anup Pandey, A. J. Ramirez-Cuesta, Adam C. Lamb, Mihail Atanasov, Eckhard Bill, Frank Neese, Zi-Ling Xue

**Affiliations:** 1 Department of Chemistry, University of Tennessee, Knoxville, TN 37996 USA; 2 Department of Physics, Florida A&M University, Tallahassee, FL 32307 USA; 3 National High Magnetic Field Laboratory, Tallahassee, FL 32310 USA; 4 Chemical and Engineering Materials Division, Spallation Neutron Source, Oak Ridge National Laboratory, Oak Ridge, TN 37830 USA; 5 Center for Neutron Research, National Institute of Standards and Technology, Gaithersburg, MD 20899 USA; 6 Max Planck Institute for Coal Research, Kaiser-Wilhelm-Platz 1, D-45470 Mülheim an der Ruhr, Germany; 7 Institute of General and Inorganic Chemistry, Bulgarian Academy of Sciences, 1113 Sofia, Bulgaria; 8 Max Planck Institute for Chemical Energy Conversion, Stiftstraße 34-36, D-45470 Mülheim an der Ruhr, Germany

## Abstract

Spin–phonon coupling plays an important role in single-molecule magnets and molecular qubits. However, there have been few detailed studies of its nature. Here, we show for the first time distinct couplings of *g* phonons of Co^II^(acac)_2_(H_2_O)_2_ (acac = acetylacetonate) and its deuterated analogs with zero-field-split, excited magnetic/spin levels (Kramers doublet (KD)) of the *S* = 3/2 electronic ground state. The couplings are observed as avoided crossings in magnetic-field-dependent Raman spectra with coupling constants of 1–2 cm^−1^. Far-IR spectra reveal the magnetic-dipole-allowed, inter-KD transition, shifting to higher energy with increasing field. Density functional theory calculations are used to rationalize energies and symmetries of the phonons. A vibronic coupling model, supported by electronic structure calculations, is proposed to rationalize the behavior of the coupled Raman peaks. This work spectroscopically reveals and quantitates the spin–phonon couplings in typical transition metal complexes and sheds light on the origin of the spin–phonon entanglement.

## Abstract (web summary)

Transition metal complexes that display slow magnetic relaxation show promise for information storage, but our mechanistic understanding of the magnetic relaxation of such compounds remains limited. Here, the authors spectroscopically and computationally characterize the strength of spin–phonon couplings, which play an important role in the relaxation process.

## Introduction

Transition metal complexes displaying slow magnetic relaxation are of great interest for possible use as single-molecule magnets (SMMs) and qubits^1–10^. One current focus is to decrease the molecular size to a single metal center^2,4,11^. To increase magnetic relaxation times, scientists have sought bistable complexes with large axial anisotropy^1–7^ and large energy barriers for the magnetization reversal^12–14^. This is usually achieved by aiming for large, negative axial zero-field splitting (ZFS) (|*D*| ≫ *kT*) and vanishing rhombicity, *E*/*D*, rendering pure *M*_*S*_ functions and no direct magnetic-dipole transitions such as *M*_*S*_ = −3/2 → +3/2 (*S* = 3/2 and *D* *<* 0). However, Gómez-Coca and coworkers recently reported that **1**, a Kramers ion with large rhombic ZFS and significant *g* anisotropy, behaves as an SMM in external magnetic fields (*D'* = (*D*^2^ + 3*E*^2^)^1/2^ ≈ 57 cm^−1^, *E*/*D* = 0.31)^15^.

Direct determination of large magnetic level separations (ZFS > 33 cm^−1^) is a challenge^4^. Phonons are prevalent in the >15 cm^−1^ region, making it difficult to distinguish them from magnetic peaks by IR or microwave spectroscopy^16^. Frequency-domain-Fourier-transform-terahertz-EPR spectroscopy (FD-FT-THz-EPR) has been used to detect 10–200 cm^−1^ magnetic gaps^10,17^. Far-IR spectroscopy has also been used to directly determine ZFS parameters^18–24^, including the recent works by van Slageren and coworkers utilizing variable magnetic fields to identify magnetic peaks in SMMs^21,22,25^.

Raman spectroscopy is seldom used to examine ZFS of transition metal complexes. In 1991, Gnezdilov and coworkers reported observation of ZFS transitions in [Fe^II^(H_2_O)_6_]SiF_6_ by Raman spectroscopy in magnetic fields^4,26^. These results agree well with those from far-IR (*D* = 11.78 cm^-1^)^27^, high-frequency electron paramagnetic resonance^28^ and frequency-domain-magnetic-resonance spectroscopies^28^. The authors attributed the Raman peaks in [Fe^II^(H_2_O)_6_]^2+^ to the presence of orbital angular momentum in the ZFS states. To the best of our knowledge, Raman spectroscopy has not been used to probe molecular magnetism in other complexes, although electronic transitions have been probed^29–35^.

Spin–phonon coupling is often the mechanism of magnetic relaxation in SMMs and qubits^1–9,36^. However, there is little understanding of these interactions, including their nature and magnitude. Phonons of SMM crystals include both intramolecular (or molecular) and lattice vibrations^37^. Recently, there has been a drive using theoretical models^38–40^ to understand how phonons lead to relaxations in SMMs. Goodwin and coworkers have reported that [Dy(Cp^ttt^)_2_][B(C_6_F_5_)_4_] (Cp^ttt^ = 1,2,4-^*t*^Bu_3_C_5_H_2_) displays magnetic hysteresis up to 60 K^40^. The magnetic relaxation is attributed to displacements primarily involving the C–H motions on the Cp^ttt^ rings. A combination of experimental methods is needed to directly observe, and thus help understand, how phonons interact with unpaired electron spins. Recent experimental evidence in this area includes work performed by Rechkemmer and coworkers to observe spin–phonon couplings of two field-dependent absorptions of a Co^II^ SMM with far-IR spectroscopy^22^.

We report here our studies of Co(acac)_2_(H_2_O)_2_ (**1**), Co(acac)_2_(D_2_O)_2_ (**1-*d***_**4**_) and Co(acac-*d*_7_)_2_(D_2_O) (**1-*d***_**18**_). Spin–phonon couplings have been probed by a combination of Raman and far-IR spectroscopies. With magnetic fields, the inter-Kramers transition moves and interacts with other phonons of *g* symmetry, rendering avoided crossings (coupling constants ≈ 1–2 cm^−1^). In Raman spectroscopy, phonon features of the coupled peaks are observed with applied magnetic fields. Far-IR spectroscopy reveals directly magnetic features of these coupled peaks. Periodic density functional theory (DFT) calculations give computed energies, atomic displacements and symmetries of the phonons in **1-*d***_**4**_ and **1-*d***_**18**_ crystals. A vibronic model has been developed for the field-dependent Raman transitions in **1**. In addition, ab initio calculations of the electronic structure in **1** reveal the origin of its ZFS.

## Results

### Structure and magnetic properties

**Fig. 1:**
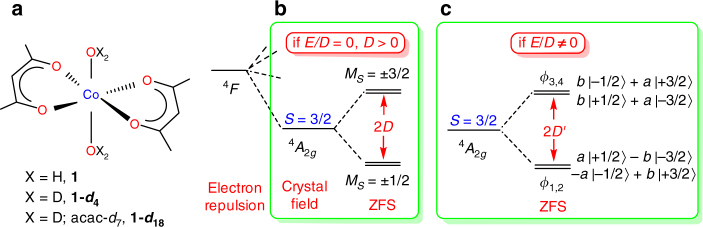
**1**, **1*-*d**_**4**_ and **1*-*d**_**18**_ and their ZFS. **a** Structures of **1**, **1-*d***_**4**_ and **1-*d***_**18**_. **b** Ground-state quartet levels in high-spin, d^7^ complexes with *D*_4*h*_ symmetry (*D* > 0; *E*/*D* = 0). **c** The quartet levels in **1** with lower symmetry [*E*/*D* ≠ 0, *D′* *=* (*D*^2^ + 3*E*^2^)^1/2^], where the mixing coefficients *a* = cos β and *b* = sin β are described by the mixing angle β obtained from the spin-Hamiltonian (*S* = 3/2) with large *D* in the absence of field^61,62^. Mixing depends on the rhombicity as tan 2β = √3 (*E*/*D*) (SI of ref. ^15^)

Compound **1** is a high-spin, d^7^ hexacoordinated Co^II^ complex with a pseudo-tetragonal structure (Fig. 1a). Its crystal structure, determined by single-crystal X-ray diffraction at 100 K, shows *C*_2*h*_ molecular symmetry with equatorial and axial Co–O distances of 2.034, 2.040 and 2.157 Å, respectively. Crystal structure of **1-*d***_**18**_ determined by powder neutron diffraction at 4 K allows the unambiguous location of D atoms (Supplementary Figs. 1–2, Supplementary Table 1 and Supplementary Note 1). If the local symmetry around the Co^II^ ion is approximated to *D*_4*h*_, the ground electronic state is ^4^*A*_2*g*_ (^4^*A*_*g*_ for *C*_2*h*_). For high-spin, d^7^ complexes in *D*_4*h*_ symmetry, ZFS leads to two Kramers doublets (KDs) that, in the absence of rhombicity in zero field, can be labeled by *M*_*S*_ = ±1/2 and ±3/2. When *D* < 0, *E*/*D* ≈ 0, the *M*_*S*_ = ±3/2 KD is the ground state with an easy axis of magnetization along the *z*-direction. For sufficiently large |*D*|, fields up to a few Tesla cannot mix the two KDs and induce any measurable magnetization in the *x*- or *y*-directions. In contrast, for *D* > 0 and *E*/*D* ≈ 0 complexes (Fig. 1b), the ground state KD *M*_*S*_ = ±1/2 is split into *M*_*S*_ = −1/2 and +1/2 states by Zeeman splitting which is strongly direction-dependent. SMM behaviors in such complexes are not expected because transitions between these two states are spin-allowed. Gómez-Coca and coworkers have shown that **1** behaves as an SMM (in external DC fields) despite its lower symmetry and dominating large rhombicity observed in EPR^15^. Magnetic susceptibility fittings revealed large ZFS *(D′* = (*D*^2^ + 3*E*^2^)^1/2^ ≈ 57 cm^−1^)^15^. EPR spectra showed typical rhombic effective *g* values (2.65, 6.95, 1.83), rendering an easy axis of magnetization (along *y*), but this is far from the usual axial situation encountered for *D* < 0, *E*/*D* ≈ 0, namely *g*′ = (0, 0, *g′*_*z*_). The best global parametrization for EPR and susceptibility data was favored to have large rhombicity, *E*/*D* = 0.31, and moderate *g* anisotropy (for *S* = 3/2, *g* = (2.50, 2.57, 2.40)). But in principle almost any value of *E*/*D* could be adopted, if the anisotropy of *g* is increased^15^. The effects are covariant, because both rhombicity and *g* anisotropy are mixing *M*_*S*_ functions, at least for finite fields, as visualized in Fig. 1c. Spin-Hamiltonian (SH) parameters cannot be deduced experimentally because no EPR spectrum is feasible for such highly excited *M*_*S*_ = ±3/2 KD in **1**. Ab initio calculations yielded different values: *D* = 91.2, *E* = 10.1 cm^−1^ (CASSCF) and *D* = 63.3, *E* = 9.3 cm^−1^ (CASPT2)^15^.

We chose **1** in part for the fact that it displays slow magnetic relaxation with *E*/*D* ≠ 0, its reported magnetic separation 2*D′ *≈ 114 cm^−1^ is relatively large and posed a challenge to measure spectroscopically, and deuterated **1-*d***_**4**_ and per-deuterated **1-*d***_**18**_ could be prepared^41^.

Typical ZFS transitions between KDs in **1** (e.g., *M*_*S*_ = −1/2 → −3/2) are magnetic-dipole-allowed by both symmetry and selection rules (Δ*M*_*S*_ = 0, ±1)^42,43^. (In the double group *D*_4*h*_′, *M*_*S*_ = ±1/2 and ±3/2 KDs are represented by *E*_1/2,*g*_$\left( {\Gamma _6^ + } \right)$ and *E*_3/2,*g*_$\left( {\Gamma _7^ + } \right)$, respectively^44,45^.) These transitions are therefore far-IR active^14,18,19^. (In the point groups *D*_4*h*_ and *C*_2*h*_, the magnetic dipole moment operators have the *E*_g_, *A*_2g_ and 2*B*_g_, *A*_g_ symmetries, respectively, as the rotations, *R*_*x*_, *R*_*y*_ and *R*_*z*_.) The “*M*_*S*_ = −1/2 → +3/2” transition is ordinarily forbidden (Δ*M*_*S*_ = 2). As discussed below, the large rhombic *E* value in **1** makes the *M*_*S*_ = +3/2 state contain the *M*_*S*_ = −1/2 character, thus rendering the “*M*_*S*_ = −1/2 → +3/2” transition magnetic-dipole-allowed. In other words, both “*M*_*S*_ = −1/2 → −3/2” and “*M*_*S*_ = −1/2 → +3/2” transitions in **1** are far-IR active. As vibronic analyses below demonstrate spin–phonon couplings of the ZFS transition with *g* phonons make the two coupled peaks contain both magnetic and phonon features. In Raman spectra, the phonon excitations of the coupled peaks reveal spin–phonon couplings in variable magnetic fields. Far-IR spectra show directly the magnetic features of the coupled peaks.

### Spin–phonon couplings in Raman spectroscopy

**Fig. 2:**
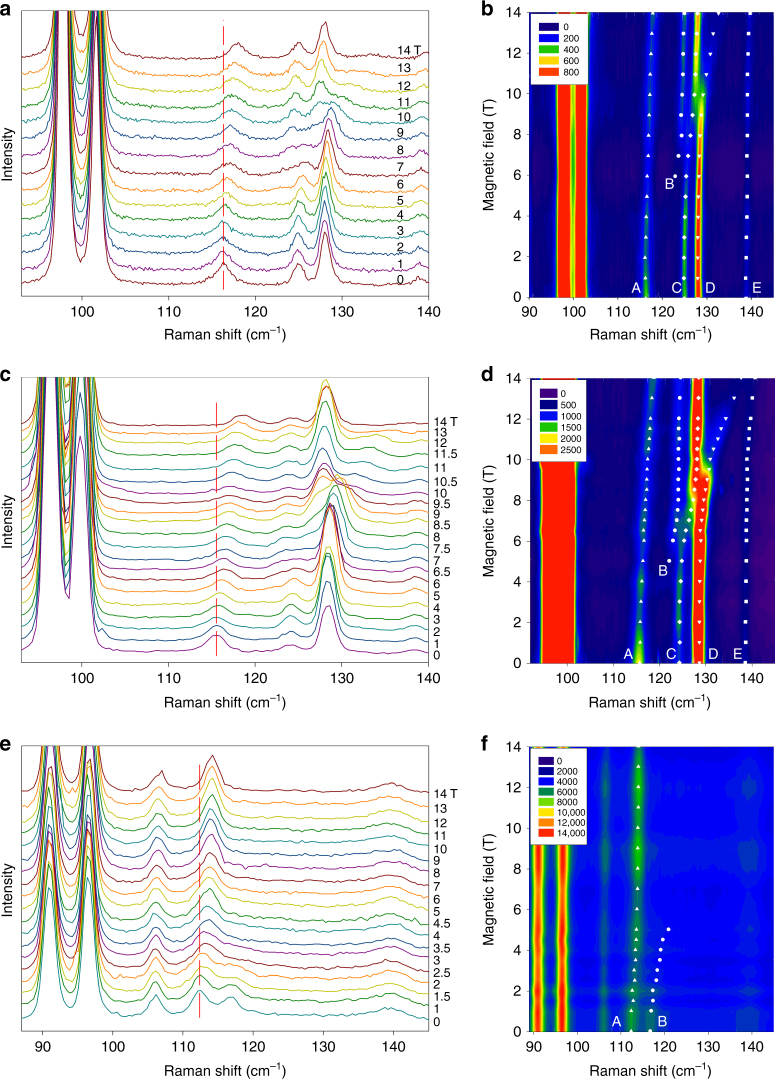
Raman spectra and contour maps in 0–14 T magnetic fields. **a**, **b** **1**, **c**, **d** **1-*d***_**4**_ and **e**, **f** **1-*d***_**18**_. Vertical lines indicate Peak A as one spin–phonon coupled peak in each set of spectra. The contour maps more clearly show the avoided crossings as a result of the spin–phonon couplings. The color codes in **b**, **d**, **f** are in units of counts. Raman spectra of **1** were collected up to 18 T but were trimmed to be consistent with other data sets

**Fig. 3:**
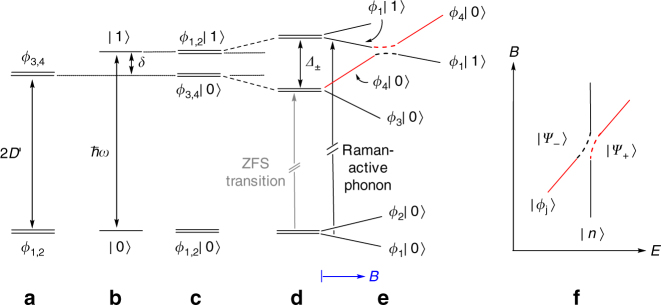
Schematic view of the spin–phonon coupling. **a** Zero-field splitting 2*D'* of the magnetic/spin quartet ground state (with eigenfunctions in Fig. 1c when no field is applied; off-axis field induces additional *M*_*S*_ mixing). **b** Vibrational states of a selected phonon with eigenfunctions |0〉 and |1〉 and a small energy separation *δ* above the excited KD *ϕ*_3,4_. **c** Spin–phonon product states with product functions *ϕ*_*i*_|*n*〉 still without vibronic coupling. **d** Vibronic coupling with coupling constant *Λ*, leading to an energy shift and splitting: *Δ*_±_ = (*δ*^2^ + *Λ*^2^)^1/2^. The ZFS transition (in gray color) is vanishingly weak in Raman spectra because it is only magnetic-dipole-allowed. **e** Zeeman splitting of vibronic states in a field *B* and avoided crossing from the coupling between the *ϕ*_4_|0〉 and *ϕ*_1_|1〉 states. Note the states *ϕ*_1,2_|0〉 and *ϕ*_1,2_|1〉 have pairwise identical slopes, whereas *ϕ*_1,2_|0〉 and *ϕ*_3,4_|0〉 have different slopes. The net transition from the lowest level *ϕ*_1_|0〉 to *ϕ*_1_|1〉 is in essence a phonon excitation and thus Raman-allowed (black arrow), and it is field-independent. When *ϕ*_4_|0〉, the upper magnetic level of the excited electronic KD, approaches *ϕ*_1_|1〉, additional coupling occurs, leading to a field-dependent transition. The ZFS transitions in (**d**) are vanishingly weak in Raman spectra, because they are only magnetic-dipole-allowed. The same holds for the *ϕ*_1_|0〉 → *ϕ*_3_|0〉, *ϕ*_1_|0〉 → *ϕ*_4_|0〉, and *ϕ*_1_|0〉 → *ϕ*_2_|1〉 transitions, which are not marked in (**e**). (Transitions from the first excited level, *ϕ*_2_|0〉, are neglected because of vanishing thermal population at 1.5–5 K.) **f** Avoided crossing in the Raman spectra based on Eq. (2). The red branches are weak in Raman intensity and only partially visible because they represent quasi-pure magnetic-dipole transitions

Raman spectra of **1**, **1-*d***_**4**_ and **1-*d***_**18**_ under 0–14 T fields are given in Figs. 2a–f. Figs. 2a-b (**1**) show four Raman peaks in the energy range 110–150 cm^−1^, which are close to the energy estimated for the excited KD at 2*D*′ ≈ 114 cm^−1^ ^15^. Interestingly, peak A at 116 cm^−1^, which is the closest to 2*D'*, is found to be slightly field-dependent, shifting monotonously to 119 cm^−1^ at 14 T. Although this feature suggests a magnetic contribution, it is unlikely to be the ZFS transition between *ϕ*_1,2_ and *ϕ*_3,4_ levels of the KDs of **1** at zero field (Figs. 1 and 3). The peak does not show Zeeman splitting and the shift rate of ~0.23 cm^−1^/T corresponds to a very small difference of effective *g* values, Δ*g*′ ≈ 0.5 (*µ*_B_ = 0.4668 cm^−1^/T). We therefore infer that peak A is predominantly of phonon origin, and its change with field reflects the magnetic feature of the spin–phonon coupled peak. At 14 T, the phonon peak is still weakly coupled to the ZFS transition. Even more interesting is that peak C at 125 cm^−1^ is field-independent below 4 T, but then attenuates with increasing field and shifts to higher energies, whereas in the same field range (4–8 T), peak B appears at ~120 cm^−1^, gaining intensity with rising field and shifting to higher energy. Above ~8 T, peak B becomes field-independent just at the energy of the weak-field branch of peak C. This behavior has the appearance of an avoided crossing. Below, we will explain the effect by coupling of a phonon at 125 cm^−1^ to the transition from the ground level *ϕ*_1_|0〉 to the excited level *ϕ*_4_|0〉, which is shifted by Zeeman effect across the phonon range (Fig. 3). In this picture, the low-field branch of peak B is Raman-silent, as it is primarily a magnetic transition when the *ϕ*_4_|0〉 level is far from the phonon energy. However, it gains intensity at 4–8 T due to mixing of the phonon with the magnetic wavefunction. The high-field branch of B is a nearly pure phonon again (at 125 cm^−1^). The shifting magnetic level at higher fields then generates a second avoided crossing with phonon peak D via the same mechanism.

Raman spectra of **1-*d***_**4**_ (Figs. 2c-d) also exhibit spin–phonon couplings similar to those of **1**, suggesting that deuteration of the water ligands in **1-*d***_**4**_ does not significantly alter magnetic peaks, phonons or their couplings in this region (110–140 cm^−1^).

In Raman spectra of **1-*d***_**18**_ (Figs. 2e-f), further deuteration has shifted many phonons compared to those of **1**/**1-*d***_**4**_. Phonon A and magnetic peak B appear to be coupled more strongly in **1-*d***_**18**_ than in **1**/**1-*d***_**4**_, such that both coupled peaks are observed at 0 T. With an applied field, A shifts to higher energy, eventually residing at 115 cm^−1^ by 6 T. B loses intensity as it shifts at the rate of ∼0.95 cm^−1^/T and vanishes by 4 T, as there are no additional *g* phonons to couple with at 120–140 cm^−1^ and 4–14 T (Fig. 2e).

Raman peak positions in magnetic fields in Figs. 2a-f are listed in Supplementary Table 2. The phonons that are coupled with the ZFS peak at 0 T, forming A and B in the spectra of **1**, **1-*d***_**4**_ and **1-*d***_**18**_, are Raman-active. In the *C*_2*h*_ group, these phonons have *A*_g_/*B*_g_ symmetry, as periodic DFT-VASP phonon calculations have shown in Supplementary Table 3.

### Spin–phonon couplings and a vibronic model for the Raman spectra

The field-driven avoided crossings in the Raman spectra can be characterized by Fig. 3^46^. A simplified Hamiltonian for the coupling between magnetic |*ϕ*_*j*_〉 and phonon |*n*〉 states (Fig. 3f) is given by the following 2 × 2 matrix Eq. (1):

1 $$H = \left( {\begin{array}{*{20}{c}} {E_{\mathrm{sp}}} & \Lambda \\ \Lambda & {E_{\mathrm{ph}}} \end{array}} \right),$$

where *E*_sp_ and *E*_ph_ are the expected energies of the magnetic and phonon excitations, respectively; *Λ* is the spin–phonon coupling constant. The energy gap between the two excited states *E*_ph_ – *E*_sp_ is *δ* (Fig. 3) which is not explicitly included in Eq. (1).

Solving the matrix gives two eigenvalues *E*_±_ (with the associated avoided-crossing peaks |*Ψ*_±_〉) in the secular Eq. (2). An alternative, detailed expression of Eq. (2) is given in Supplementary Note 2. Considering that Eq. (2) involves *Λ*^2^, the sign of *Λ* may not be determined from the Raman spectra here.

2 $$\left| {\begin{array}{*{20}{c}} {E_{{\mathrm{sp}}}-E}_{\pm} & \Lambda \\ \Lambda & {E_{\mathrm{ph}} - E}_{\pm} \end{array}} \right| = 0.$$

Upon coupling, |*Ψ*_+_〉 shifts to higher *E*_+_ while |*Ψ*_−_〉 shifts to lower *E*_*−*_, as shown in Fig. 3f ^46^. For example, both states |*Ψ*_±_〉, giving rise to peaks A and B in the Raman spectra of **1-*d***_**18**_ at 0 T (Figs. 2e-f), contain magnetic and phonon features (Fig. 3). Since the phonon here is Raman-active, the phonon portions of both A and B make the two peaks observable in the Raman spectra.

Eqs. (1–2) provide a model to understand the spin–phonon couplings in the Raman spectra (Figs. 2a–f) and calculate the coupling constants, as discussed below. However, it should be pointed out that for the Hamiltonian in Eq. (1), vibronic coupling in the ground KD is neglected. In principle, however, both the ground and excited KD states are involved in a transition, each has a spin and vibrational substate, which all may interact with each other. Thus, a more complete Hamiltonian should be at least a 6 × 6, or better, an 8 × 8 matrix. In contrast, Eq. (1) assumes that the ground KD state is not involved in spin–phonon coupling. In addition, this simple model assumes weak spin–phonon couplings. Therefore, terms higher than single phonon excitations are neglected. A more precise vibronic model for the spin–phonon couplings is presented in Methods, Supplementary Figs. 3–5 and in Supplementary Notes 3–4 and will be discussed below. Lastly, this model only considers coupling between the magnetic transition and one phonon, typically the phonon closest in energy to the ZFS transition. However, other distant *g* phonons may also be coupled to the magnetic transition, although weakly, thus taking the magnetic feature away.

**Fig. 4:**
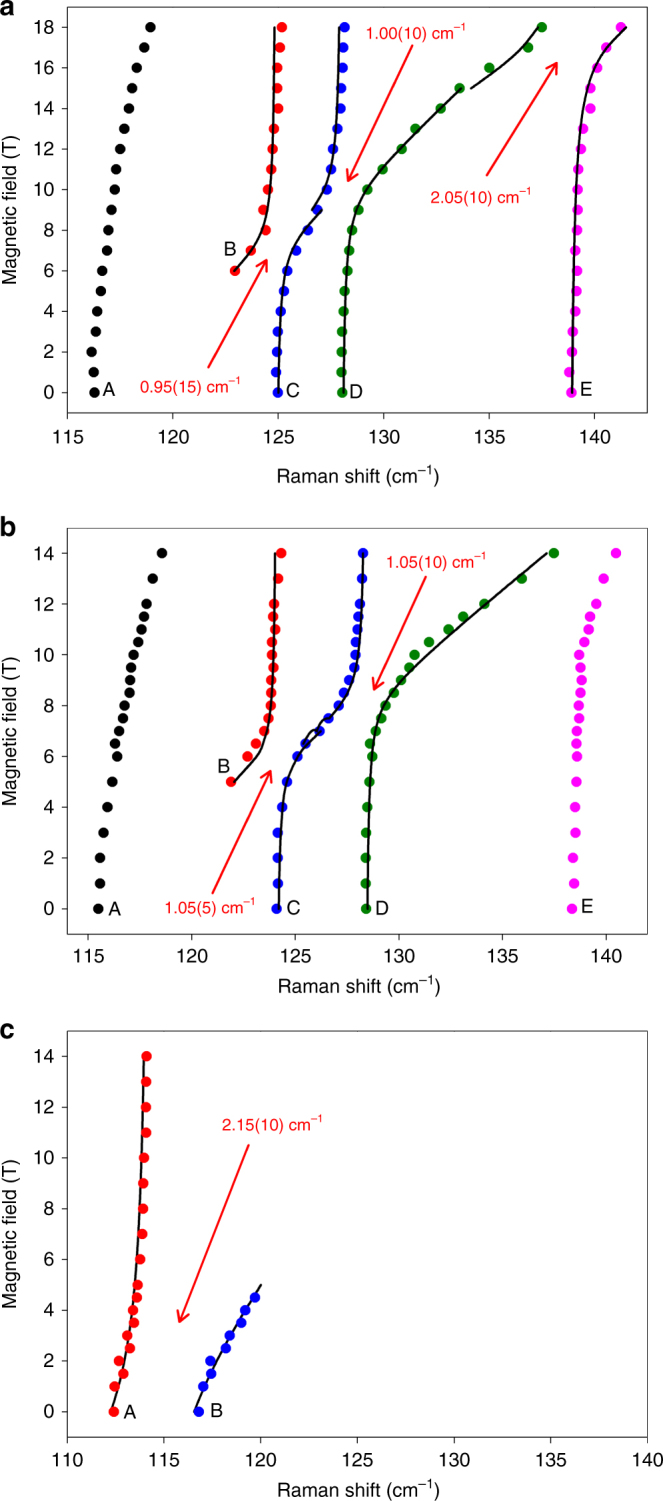
Peak positions vs. magnetic fields for selected transitions in the Raman spectra. **a** **1**; **b** **1-*d***_**4**_; **c** **1-*d***_**18**_. The solid lines are fittings using Eq. (2), giving the coupling constants |*Λ*|. Arrows point to corresponding avoided crossings for |*Λ*|

Using Eq. (2) to fit the spin–phonon couplings in Figs. 2a–f yields the coupling constants |*Λ*| for each avoided crossing (Fig. 4). |*Λ*| corresponds to roughly half the distance between the peaks at their closest positions. The larger the coupling constant, the greater their repulsion (Fig. 4).

**Fig. 5:**
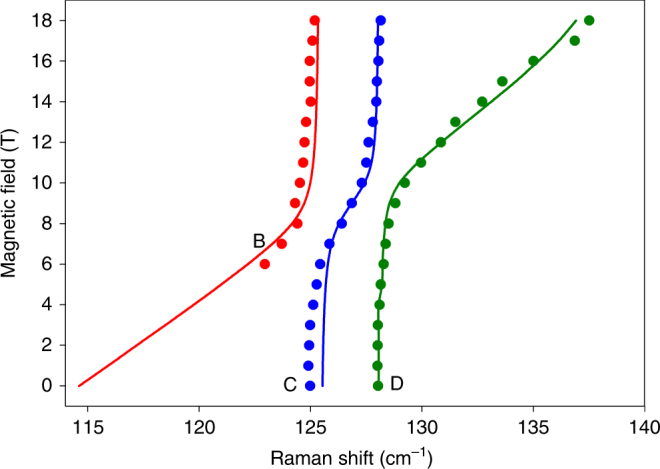
Fitting of the Raman spectra. Simulated (solid lines) and experimental (circles) positions of field-dependent (*B*||*z*) Raman transitions corresponding to peaks B (red), C (blue) and D (green) of **1**. *g*_z_ = 1.49, *ħω*_1_ = 125.4 cm^−1^, *ħω*_2_ = 128.1 cm^−1^, *ħω*_3_ = 139.5 cm^−1^ (not shown in the figure); *E*_1_ = 1.14 cm^−1^, *E*_2_ = 0.88 cm^−1^, *E*_3_ = 2.66 cm^−1^ [*E*/*D* = 0.17 (ORCA NEVPT2); 2*D'* = 115 cm^−1^]. *E*_1_, *E*_2_ and *E*_3_ from the vibronic calculations are the coupling constants |*Λ*_1_|, |*Λ*_2_| and |*Λ*_3_|, Eq. (2), respectively, for the interaction with the *ħω*_1_, *ħω*_2_ and *ħω*_3_ modes. Simulated and experimental positions of field-dependent (*B*||*x* and *B*||*y*) Raman transitions are given in Supplementary Fig. 4

We have developed a more detailed vibronic model to quantify the spin–phonon couplings in Fig. 4. Complex **1** possesses a large rhombicity *E*/*D*. Parameters of the vibronic coupling model, extracted from the experimental field-dependent Raman spectra, turn out to be rather insensitive to the *E*/*D* ratio (Supplementary Table 4). Thus, we base qualitative discussions using our model on *E* = 0. Magnetic-field-dependent Raman spectra of **1** (Figs. 2a-b) consist of five branches A–E. For branches C–E, the regions at low and high fields show almost no field dependence. While not observed at low fields, B displays no field dependence at high field. A and C–E correspond to vibrations with estimated zero-field energies of *ħω*_0_ = 116, *ħω*_1_ = 125, *ħω*_2_ = 128 and *ħω*_3_ = 139 cm^−1^. At intermediate magnetic fields, branches B–D display the slope of a magnetic-field-induced spin-transition as avoided crossings. There are three avoided crossing points between B–C, C–D and D–E at 7.64, 9.43 and 17.54 T with energies 125.05, 127.99 and 138.71 cm^−1^, respectively (Supplementary Table 5). Here, magnetic excitations from the ground into the excited level would appear when no crossing (|*Λ*| = 0) is present. Energies of these unseen magnetic excitations increase with field and cross the three different vibrational levels (0 T) at *ħω*_1_ = 125 (C), *ħω*_2_ = 128 (D) and *ħω*_3_ = 139 (E) cm^−1^. *ħω*_1–3_ are energies at the crossing points (1/2(*E*_sp_ + *E*_ph_), Eq. (2)) from the B–C, C–D and D–E couplings, respectively. Fig. 5 displays simulations of the Raman transitions in the *B*||*z* field. The *B*||*x* and *B*||*y* field directions were fitted as well, but neither was a close match to the experimental results (Supplementary Fig. 4). Analyses of the field-dependent Raman peaks were performed to potentially determine *E*/*D*. However, results of the analyses indicate that the derived parameters (Supplementary Table 4) are mostly insensitive to *E*/*D*. Discussions of the mechanism of the intensities in the field-dependent Raman spectra are given in Supplementary Note 5 and Supplementary Figs. 6–7.

To the best of our knowledge, these are the first direct observation of spin–phonon couplings (as avoided crossings) in Raman spectra of a molecular compound and their quantification. Brinzari and coworkers have studied ferromagnetic, MOF (metal-organic framework)-like Co^II^[N(CN)_2_]_2_ and also found a phonon-coupled, field-dependent transition in Raman spectra^47^.

### Spin–phonon couplings in far-IR spectroscopy

**Fig. 6:**
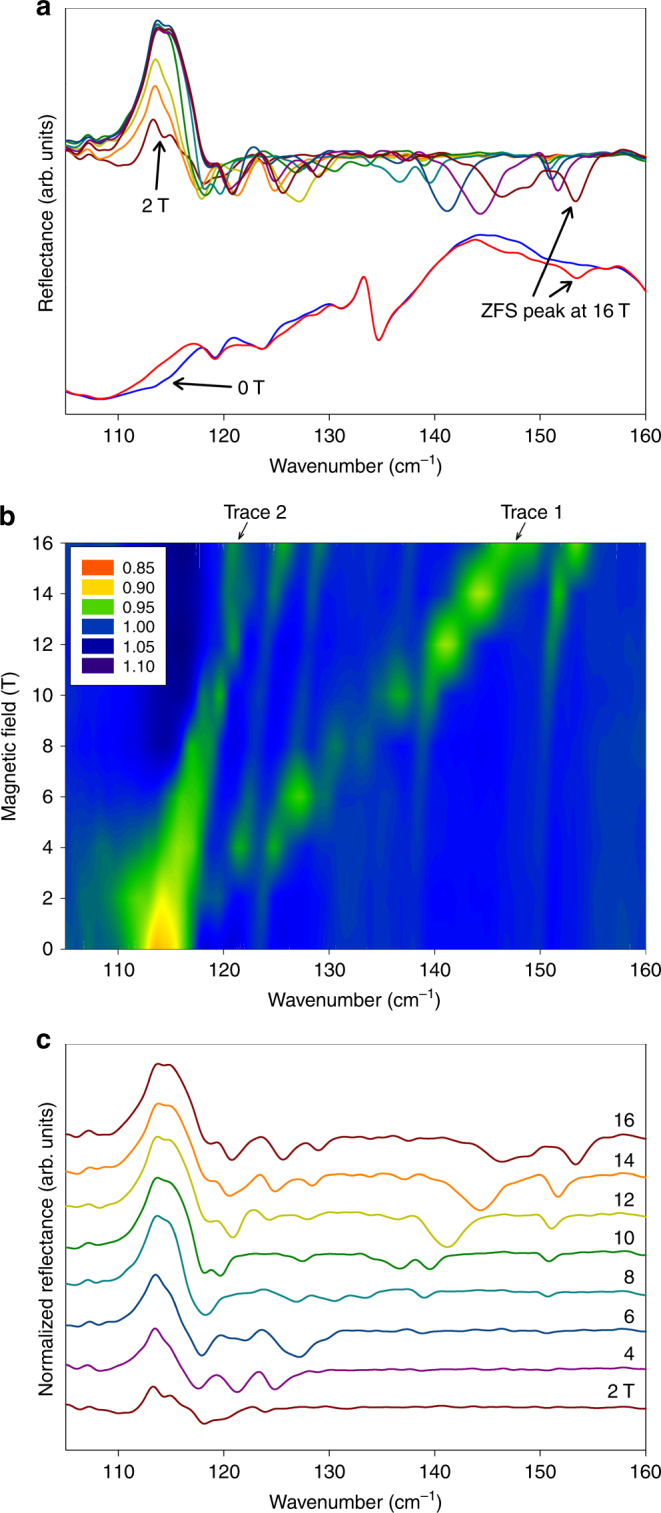
Far-IR reflectance spectra of **1*-*d**_**4**_. **a** Reflectance spectra (bottom, 0 T-blue and 16 T-red) and normalized (by the 0 T) reflectance spectra (top) by a single crystal of **1-*d***_**4**_, in which the ZFS transition is more visible as it shifts with field at 2, 4, 6, 8, 10, 12, 14 and 16 T. **b** Contour plot of the normalized reflectance spectra (by the average of all spectra) which shows the ZFS transitions and the magnetic features of spin–phonon coupled peaks. **c** Normalized reflectance spectra using data in **a**

As discussed earlier^14,18,19^, transitions between the two KDs are in general magnetic-dipole-allowed and therefore are potentially far-IR active. For the spin–phonon coupled states of **1**, **1-*d***_**4**_ and **1-*d***_**18**_ in Fig. 3, the magnetic features of the transitions are far-IR active. In a diffuse reflectance measurement of a single crystal of **1-*d***_**4**_ (Fig. 6), the most significant difference between spectra of 0 and 16 T fields is a loss in absorption at ~115 cm^−1^ (Fig. 6a). Normalizing these spectra (by dividing them by the 0 T spectrum to remove field-independent absorptions) reveals additional details (Fig. 6a) which are further enhanced in a color-coded contour plot (Fig. 6b).

**Fig. 7:**
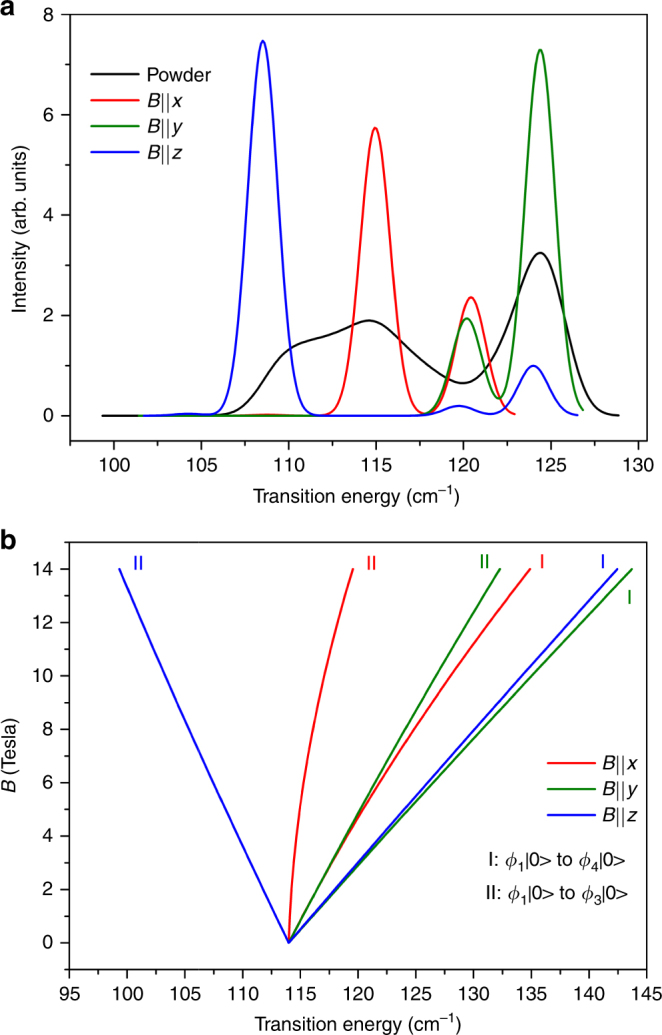
Simulated inter-KD, magnetic-dipole-allowed transitions and their field dependence. **a** Simulated inter-KD, magnetic-dipole-allowed transitions for the *S* = 3/2, spin-only Hamiltonian description of **1**. Single-crystal spectra for fields of 5 T in the *x, y, z* directions at 1.7 K are shown in red, green and blue. The powder average is given in black. Vibronic coupling was ignored. Spin-Hamiltonian parameters are taken from ref. ^15^: *D* = 57 cm^−1^, *E*/*D* = 0.31, *g*_*x*_ = 2.50, *g*_*y*_ = 2.57, *g*_*z*_ = 2.40 (corresponding *g'*-values for ground and excited KDs: *g'*_*i*_(1) = 2.65, 6.95, 1.83; and *g'*_*i*_(2) = 2.34, 1.80, 6.63, respectively). The dominant pairs of left and right lines found for the single-crystal orientations (in blue, green, red) correspond to the dominant *ϕ*_1_|0〉 → *ϕ*_3_|0〉 and *ϕ*_1_|0〉 → *ϕ*_4_|0〉 transitions, respectively. **b** Field dependence of the inter-KD spectra for fields in the *x, y, z* directions

The most remarkable feature is a (weak) field-dependent absorption, moving from 114 cm^−1^ at 0 T to ~150 cm^−1^ at 16 T (trace 1, Fig. 6b). The shift rate of 2.25 cm^−1^/T reveals a difference (or sum) of *g*′ values of the initial and final levels of Δ*g*′ ≈ 4.8. From a comparison with the principal *g*′ values obtained from the previous spin-Hamiltonian parametrization for **1**^15^(*g*′_*i*_(1,2) = 2.65, 6.95, 1.83 for *ϕ*_1,2_ of the lower KD and *g'*_*i*_(3,4) = 2.34, 1.80, 6.63 for *ϕ*_3,4_ of the excited KD), we can infer in first order that the main observed field-dependent IR-peak (trace 1) may be from one of two possible transitions. The first is the *ϕ*_1_|0〉 → *ϕ*_3_|0〉 transition with the field in the *y*-direction (Δ*g'* = 6.95 - 1.80 = 5.15; green line II in Fig. 7b); The second is the *ϕ*_1_|0〉 → *ϕ*_4_|0〉 transition with the field in the *x*-direction (sum of *g'* values: 2.65 + 2.34 = 4.99; red line I in Fig. 7b). (At 5 K for the far-IR studies, only the *ϕ*_1_|0〉 should be thermally populated, at least for moderate to strong fields.) Corresponding simulations, using the full spin-Hamiltonian (*S* = 3/2) for the three principal field orientations (*B*||*x, B*||*y, B*||*z*) and for both magnetic transitions to the excited KD, are given in Fig. 7. If trace 1 is the *ϕ*_1_|0〉 → *ϕ*_3_|0〉 transition (green line II in Fig. 7b) with the field in the *y*-direction (first possible transition above), another transition (green line I) to the right of trace 1 would be expected. However, no such trace is obvious in Fig. 6b, suggesting that trace 1 is unlikely the *ϕ*_1_|0〉 → *ϕ*_3_|0〉 transition with the field in the *y*-direction. If trace 1 is the *ϕ*_1_|0〉 → *ϕ*_4_|0〉 transition (red line I in Fig. 7b) with the field in the *x*-direction (second possible transition above), the *ϕ*_1_|0〉 → *ϕ*_3_|0〉 transition in the *x*-direction (red line II in Fig. 7b) to the left of trace 1 in Fig. 6b is expected. Such behavior can be explained by the difference in the effective *g* values, Δ*g'* = 2.65 − 2.34 = 0.31, which is still positive. In fact, traces 1 and 2 in Fig. 6b are consistent with the analysis. Starting around 114 cm^−1^ at *B* = 0, traces 1 and 2 are the *ϕ*_1_|0〉 → *ϕ*_4_|0〉 (red line I) and *ϕ*_1_|0〉 → *ϕ*_3_|0〉 (red line II) transitions, respectively. However, it should be noted that any such assignment is a simplification when the crystal orientation is not known, because other, off-axis orientations of the field may yield similar results.

Spin–phonon coupling, which was not included in the above analysis of Fig. 6, should not change the general picture. However, it may explain the “gaps” observed in the field-dependence of the *ϕ*_1_|0〉 → *ϕ*_4_|0〉 transition (trace 1). We suggest that the mixing of the *ϕ*_4_|0〉 state with IR-silent *g* phonons at the points of the avoided crossings reduces the absorption probability by 50%. As a result, rather sharp, distinct gaps occur for the magnetic transition (trace 1) at the phonon energies, as nicely observed around *ħω*_1_ = 125, *ħω*_2_ = 128 and *ħω*_3_ = 139 cm^−1^, which have been assigned above to the *g* phonon peaks C, D, and E in the Raman spectra (Figs. 2c-d).

Simulations in Fig. 7 support the analysis discussed earlier that both *ϕ*_1_ → *ϕ*_3_ and *ϕ*_1_ → *ϕ*_4_ inter-KD transitions in **1** are magnetic-dipole allowed and are expected to be observable in far-IR. The two transitions, each in the *x, y, z* directions inside magnetic fields, lead to the expected shifting patterns of the six lines in Fig. 7b. Most lines, except one, are blue-shifted to higher energies (Fig. 7a). Thus, average far-IR spectra of a powder sample of **1** are expected to be blue-shifted and reveal the magnetic features of the spin–phonon coupled peaks. Indeed, the transmittance far-IR spectra of **1** (Supplementary Figs. 8a–b and 9) show these features, except that the coupled peaks are not resolved as in the Raman spectra (Figs. 2a-b). The far-IR transmittance spectra of a powder sample of **1-*d***_**4**_ (Supplementary Figs. 8c–d and 10) are also consistent with the spin–phonon coupling and features of the far-IR spectra of the single crystal of **1-*d***_**4**_ (Fig. 6). Far-IR transmittance of **1-*d***_**18**_ reveals similar features in Supplementary Figs. 8e–f and 11.

Additional discussions of the far-IR spectra are given in Supplementary Note 6. In the far-IR spectra of **1-*d***_**4**_, there are four *u* phonons between 115 and 143 cm^−1^ (Supplementary Table 3). Their symmetries have been assigned by the VASP calculations discussed below. No observed coupling between these *u* phonons and the ZFS peak is found in far-IR spectra.

The results here from the Raman and far-IR spectroscopies show that only the couplings of the ZFS transition to the *g* phonons in **1**, **1-*d***_**4**_ and **1-*d***_**18**_ are observed in Raman spectra. Far-IR spectra in this work do not reveal couplings to the *u* phonons. Work on the transition matrix in the future may provide an understanding. It should be noted, however, that pattern of the couplings is limited to the current complexes. Additional work on other complexes, especially those with different symmetries, is needed to have a comprehensive understanding of the couplings.

### Periodic DFT phonon calculations and comparisons with experiments

Phonon modes for *C*_2*h*_**1-*d***_**4**_ and **1-*d***_**18**_ are calculated by VASP (Supplementary Table 3) and show atomic displacements with contributions from both external (lattice) and internal modes. In the region of interest here, ~115 cm^−1^, vibrations are not localized but involve atomic displacements of the whole molecule, as demonstrated in Supplementary Movies 1–5. The modes with the largest spin–phonon coupling constant |*Λ*|, E of **1/1-*d***_**4**_ (Supplementary Movie 4 for phonon E of **1-*d***_**4**_) and A of **1-*d***_**18**_ (Supplementary Movie 5), have greatly mismatched vector magnitudes of the equatorial O atoms, leading to a larger net change in this bond angle (Supplementary Table 6). These vibrations significantly distort the first coordination sphere and perhaps lead to the larger |*Λ*|. Therefore, we rationalize that, if these phonons are involved in magnetic relaxation, the O–Co–O equatorial-bond-angle distortion plays a key role in the spin reversal. These spin changes of the excited KD is of prime importance for the magnetic relaxation at elevated temperatures where the excited KD is populated. Likewise, low-energy phonons (not included in Supplementary Table 3) are responsible for the low-temperature shortcut of the relaxation time. These effects are beyond the scope of this work. Modes C and D of **1-*d***_**4**_ (Supplementary Movies 2 and 3 for phonons C and D, respectively) have less distortion of the O–Co–O equatorial bond angle and therefore, we reason, do not couple as strongly with spin. These findings are in line with recent calculations of spin–phonon couplings in [(tpa^Ph^)Fe]^-^ [H_3_tpa^Ph^ = tris((5-phenyl-1*H*-pyrrol-2-yl)methyl)amine] by Lunghi and coworkers demonstrating that the vibrations perturbing the bending angle of the equatorial *N* atoms coordinated to the Fe^II^ ion are strongly coupled to the spin^38^.

Additional results of the phonon calculations, including distortion of the O–Co–O bond angle in the equatorial plane compared with the spin–phonon coupling constants |*Λ*| (Supplementary Table 6), are given in Supplementary Figs. 12–13 and Supplementary Note 7. Supplementary Movie 1 for phonon A of **1-*d***_**4**_ is also provided.

### Origin of ZFS in 1 analyzed by ab initio calculations

Although **1** has been studied as a model complex^15,48,49^, its ZFS origin is not clear. Electronic structure of **1** has been reconsidered using multireference ab initio calculations in close relation and comparison with two basic experimental studies^15,49^, including the single-crystal EPR work by Bencini and coworkers^49^, in order to probe the origin. Lohr and coworkers have calculated the electronic structure of **1** with descending crystal field symmetry from octahedral to orthorhombic and used the results to obtain magnetic properties^48^. Details of the current electronic structure calculations and comparisons with experimental results are given in Supplementary Figs. 14–18, Supplementary Tables 7–8 and Supplementary Note 8.

According to the orbital energy diagram, $d_{{ x' }{ z' },{ y' }{ z' }} < d_{{ x' }^{^{ 2 }}{-}{{ y' }^{^{ 2 }}}}$(Supplementary Fig. 17), the ^4^*T*_1*g*_ state of a high-spin octahedral Co^II^ complex undergoes a *D*_4*h*_ splitting into an ^4^*A*_2*g*_ ground state and an ^4^*E*_*g*_ excited state. When the symmetry is lowered to *D*_2*h*_ and *C*_2*h*_, ^4^*E*_*g*_ (*D*_4*h*_) state undergoes further splitting. Energies of all ten *S* = 3/2 states and the effect of symmetry lowering are listed in Supplementary Table 7. The sublevels of ^4^*T*_1*g*_ are well separated from the excited ^4^*T*_2*g*_ levels with the overall splitting of the ^4^*T*_1*g*_ level about twice the effective Co^II^ spin–orbit coupling (SOC) parameter (530 cm^−1^).

Ab initio NEVPT2 calculations indicate that the splitting between the two KDs is 169.8 cm^−1^, with the SOC-excited states stemming from the ^4^*E*_g_ levels to be at 884.1, 1144.7, 1481.9, and 1616.2 cm^−1^, showing that there are no other excited states in the vicinity of the lowest excited level at 169.8 cm^−1^. The computed and *g'*_*x*_, *g'*_*y*_ and *g'*_*z*_ values of the lowest KD are 3.745, 6.846 and 1.864, respectively.

From the *D* eigenvalues, we deduce *D* and *E*, *D* = 3/2 *D*_*zz*_ = 81.4 and *E* = (*D*_*xx*_ - *D*_*yy*_)/2 = 14 cm^−1^ and *E*/*D* = 0.17. At the temperatures available to probe the magnetic properties by magnetic susceptibility, field-dependent magnetization and EPR, there is no appreciable population of the lowest excited KD state.

High-quality single EPR spectra have been deduced from a single-crystal, X-band study reporting *g* values of 2.74, 6.84 and 1.88^49^. They compare in magnitude and direction well with the computed results (vide supra). Parameters of the spin-Hamiltonian deduced from an interpretation of both the low-temperature magnetic data and the EPR spectra have been used to deduce the principal values of the gyromagnetic tensor and the ZFS^15^: *D* = 57.0, *E*/*D* = 0.31, *g*_*x*_ = 2.50, *g*_*y*_ = 2.57, *g*_*z*_ = 2.40 and *g*′_*x*_ = 2.65, *g*′_*y*_ = 6.95, *g*′_*z*_ = 1.83. They are again compatible with the computed results in Supplementary Table 8.

Current studies spectroscopically reveal and quantitate the spin–phonon couplings in a typical Kramers complex. These studies offer a unique look at how spectroscopies can be utilized to study spin–phonon couplings in molecular complexes. The work here provides a rare case to compare Raman and far-IR spectroscopies and shows how the two, working together with ab initio and periodic DFT phonon calculations, reveal the spin–phonon couplings. In addition, the vibronic model developed to understand the Raman data sheds light on the origin of spin–phonon entanglement. At different external magnetic fields, the ZFS peak couples to different phonons. The spectroscopies at magnetic fields >14 T may reveal further couplings of the ZFS transition with other phonons not observed in this work. These experiments confirm the importance of obtaining spin–phonon coupling constants to understand how the lattice promotes relaxation at elevated temperatures. Importantly, spin–phonon coupling is not exclusively a phenomenon in SMMs, but is observed in a variety of magnetic materials.

We expect that the Raman and far-IR spectroscopies could be used to probe f complexes and d complexes with the first-order orbital momentum. Electric-dipole or magnetic-dipole transitions between states may be observed in far-IR, IR, or UV–visible spectroscopies^50^. SOC is generally larger than the effect of the crystal field for f complexes^51^. States in f complexes thus have both orbital and spin features as a result of the coupling. Transitions between these states are thus also Raman-active, following the electronic Raman selection rules (Δ*J* ≤ 2, Δ*L* ≤ 2, Δ*S* = 0)^52^. This is in contrast to the current work on a d complex with quenched first-order orbital angular momentum, where the Raman peaks are phonon parts of spin–phonon coupled peaks and the spin parts are from the ZFS transition.

## Methods

### Synthesis of **1**, **1-*d***_**4**_ and **1-*d***_**18**_

The following chemicals were used as received: Co(acac)_2_ (Alfa Aesar), CoCl_2_ (Alfa Aesar), acetylacetone (Fisher Scientific), K_2_CO_3_ (Sigma-Aldrich), D_2_O (99.9% D, Cambridge Isotope Laboratories) and CH_2_Cl_2_ (Fisher Scientific, Certified ACS grade). Dimethylformamide (Fisher Scientific, Certified ACS grade) was dried using 5 Å molecular sieves.

Complex **1** was synthesized according to the method of Ellern and coworkers^53^ by dissolving the anhydrous tetramer Co(acac)_2_ in DMF and adding H_2_O to the dark purple solution. The solution lightened and pinkish-orange crystals formed. Replacing H_2_O with D_2_O yielded the partially deuterated compound **1-*d***_**4**_. Larger crystals were obtained when less H_2_O/D_2_O was used and allowed to crystallize at −35 °C.

Deuterated acetylacetone was prepared by the method of Frediani et al.^41^. Acetylacetone (10 mL, 9.8 g, 0.098 mol) was added to 100 mL of D_2_O and 1 g of K_2_CO_3_ into a Schlenk flask under nitrogen gas. The solution was refluxed under nitrogen overnight at 120 °C. After cooling the solution to room temperature, the organic product, deuterated acetylacetone, was extracted from the aqueous layer using CH_2_Cl_2_. Solvent was then removed in vacuo. Deuteration level was analyzed using DART (Direct Analysis in Real Time) mass spectrometry. The process was repeated a second time with another 100 mL of D_2_O to give acetylacetone-*d*_8_ (91% D; 100% yield).

Co(acac-*d*_7_)_2_(D_2_O)_2_ (**1-*d***_**18**_) was synthesized by mixing D_2_O (20 mL, 22 g, 1.1 mol), acetylacetone-*d*_8_ (2.5 mL, 2.4 g, 22 mmol) and CoCl_2_ (0.30 g, 2.3 mmol). K_2_CO_3_ (3.12 g) was qualitatively added to dissolve the acetylacetone-*d*_8_ until traces of an amorphous solid began to precipitate. The solution was filtered, followed by further addition of K_2_CO_3_ until polycrystalline **1-*d***_**18**_ formed. The mixture was filtered and washed with D_2_O to give **1-*d***_**18**_ (0.45 g, 62% yield based on CoCl_2_).

### Far-IR and Raman spectroscopies under variable magnetic fields

Far-IR and Raman spectroscopic studies were conducted at the National High Magnetic Field Laboratory (NHMFL) at Florida State University. For reflectance far-IR spectra of **1-*d***_**4**_, an unoriented single crystal was used. For transmittance far-IR spectra, the powdered samples were mixed with eicosane and pressed into pellets that were approximately 1 mm thick. Spectra were collected at 5 K using a Bruker Vertex 80v FT-IR spectrometer coupled with a superconducting magnet (SCM) with fields up to 17.5 T.

Raman samples were prepared with unoriented single crystals of **1** and **1-*d***_**4**_ and powders of **1-*d***_**18**_. Data were collected by a backscattering Faraday geometry using a 532 nm laser at a 14 T SCM in the Electron Magnetic Resonance (EMR) facility and an 18 T SCM in the DC Field facility. Crystals of samples were cooled at 5 K (14 T) and 1.5 K (18 T). Collected scattered light was guided via an optical fiber to a spectrometer equipped with a liquid-nitrogen-cooled CCD camera.

### Vibronic model for the magnetic-field-dependent Raman spectra of **1**

The vibronic coupling model here, an extension of that in ref. ^22^ applied for a single mode, accounts for three intervening vibrations coupling to the *M*_*S*_ = ±1/2, ±3/2 sublevels of *S* = 3/2 spin. The Hamiltonian of the spin–phonon coupled system of a spin (*S*) with three vibrations is composed of three terms representing the spin $(\hat H_S)$, the phonons $(\hat H_{{\mathrm{vib}}})$ and the spin phonon coupling $(\hat H_{S - {\mathrm{vib}}})$:

3 $$\hat H_{{\mathrm{eff}}} = \hat H_S + \hat H_{{\mathrm{vib}}} + \hat H_{S - {\mathrm{vib}}}.$$

The spin-Hamiltonian for an *S* = 3/2 spin is:

4 $$\hat H_S = D(\hat S_z^2 - 5/4) + E(\hat S_x^2 - \hat S_y^2) + \beta _Bg_xB_x\hat S_x + \beta _Bg_yB_y\hat S_y + \beta _Bg_zB_z\hat S_z,$$

For the three vibrations (*i* = 1,2,3):

5 $$\hat H_{{\mathrm{vib}}} = \mathop {\sum}\limits_i {\hbar \omega (n_i + 1/2)}$$

the spin–phonon coupling Hamiltonian is:

6 $$\hat H_{S - {\mathrm{vib}}} = \mathop {\sum}\limits_i {(\partial E/\partial Q_i)_oQ_i(\hat S_x^2 - \hat S_y^2) + (\partial D/\partial Q_i)_oQ_i(\hat S_z^2 - 5/4)}$$

With $\left| {M_S = \pm 3/2} \right\rangle$ and $\left| {M_S = \pm 1/2} \right\rangle$ as the basis functions for the spin-sublevels of the *S* = 3/2 spin and $\chi _{n_i}(Q_i),$ *i* = 1,2,3 as the harmonic oscillator wavefunctions for the three interacting modes, the spin–phonon wavefunction $\left| {\Psi _{S - {\mathrm{vib}},k}} \right\rangle$ can be expanded into a series of products as spin-sublevels and the three vibrational functions:

7.1 $$\left| {\Psi _{S - {\mathrm{vib}},k}} \right\rangle = \mathop {\sum}\limits_{M_S = \pm 1/2, \pm 3/2} {\mathop {\sum}\limits_{n_1,n_2,n_3} {c_{k,M_S,n_1,n_2,n_3}\left| {M_S} \right\rangle } } \chi _{n_1}(Q_1)\chi _{n_2}(Q_2)\chi _{n_3}(Q_3).$$

Under the assumption of a weak spin–phonon coupling, one can restrict the calculations to the ground and lowest phonon excited states: *n*_*i*_ = 0,1 leading to the following set non-vanishing product functions in the expansion of Eq. (7.1):

7.2 $$\begin{array}{l}\left| {M_S} \right\rangle \chi _{n_1}(Q_1)\chi _{n_2}(Q_2)\chi _{n_3}(Q_3):\\ \left| {3/2} \right\rangle \chi _0(Q_1)\chi _0(Q_2)\chi _0(Q_3) = (3/2,0,0,0),\end{array}$$

7.3 $$\left| {1/2} \right\rangle \chi _0(Q_1)\chi _0(Q_2)\chi _0(Q_3) = (1/2,0,0,0),$$

7.4 $$\left| { - 1/2} \right\rangle \chi _0(Q_1)\chi _0(Q_2)\chi _0(Q_3) = ( - 1/2,0,0,0),$$

7.5 $$\left| { - 3/2} \right\rangle \chi _0(Q_1)\chi _0(Q_2)\chi _0(Q_3) = ( - 3/2,0,0,0),$$

7.6 $$\left| {3/2} \right\rangle \chi _1(Q_1)\chi _0(Q_2)\chi _0(Q_3) = (3/2,1,0,0),$$

7.7 $$\left| {1/2} \right\rangle \chi _1(Q_1)\chi _0(Q_2)\chi _0(Q_3) = (1/2,1,0,0),$$

7.8 $$\left| { - 1/2} \right\rangle \chi _1(Q_1)\chi _0(Q_2)\chi _0(Q_3) = ( - 1/2,1,0,0),$$

7.9 $$\left| { - 3/2} \right\rangle \chi _1(Q_1)\chi _0(Q_2)\chi _0(Q_3) = ( - 3/2,1,0,0),$$

7.10 $$\left| {3/2} \right\rangle \chi _0(Q_1)\chi _1(Q_2)\chi _0(Q_3) = (3/2,0,1,0),$$

7.11 $$\left| {1/2} \right\rangle \chi _0(Q_1)\chi _1(Q_2)\chi _0(Q_3) = (1/2,0,1,0),$$

7.12 $$\left| { - 1/2} \right\rangle \chi _0(Q_1)\chi _1(Q_2)\chi _0(Q_3) = ( - 1/2,0,1,0),$$

7.13 $$\left| { - 3/2} \right\rangle \chi _0(Q_1)\chi _1(Q_2)\chi _0(Q_3) = ( - 3/2,0,1,0),$$

7.14 $$\left| {3/2} \right\rangle \chi _0(Q_1)\chi _0(Q_2)\chi _1(Q_3) = (3/2,0,0,1),$$

7.15 $$\left| {1/2} \right\rangle \chi _0(Q_1)\chi _0(Q_2)\chi _1(Q_3) = (1/2,0,0,1),$$

7.16 $$\left| { - 1/2} \right\rangle \chi _0(Q_1)\chi _0(Q_2)\chi _1(Q_3) = ( - 1/2,0,0,1),$$

7.17 $$\left| { - 3/2} \right\rangle \chi _0(Q_1)\chi _0(Q_2)\chi _1(Q_3) = ( - 3/2,0,0,1).$$

Within this basis, the non-vanishing matrix elements of the spin–phonon coupling Hamiltonian are given by

8 $$\left\langle { \pm \frac{3}{2},1} \right|(\partial E/\partial Q_i)_oQ_i(\hat S_x^2 - \hat S_y^2)\left| { \mp \frac{1}{2},0} \right\rangle = \sqrt {\frac{3}{2}} (\partial E/\partial Q_i)_o = E_i,$$

9 $$\left\langle { \pm \frac{3}{2},1} \right|(\partial D/\partial Q_i)_oQ_i(\hat S_z^2 - 5/4)\left| { \pm \frac{3}{2},0} \right\rangle = \frac{1}{{2\sqrt 2 }}(\partial D/\partial Q_i)_o = D_i,$$

10 $$\left\langle { \pm \frac{1}{2},1} \right|(\partial D/\partial Q_i)_oQ_i(\hat S_z^2 - 5/4)\left| { \pm \frac{1}{2},0} \right\rangle = - \frac{1}{{2\sqrt 2 }}(\partial D/\partial Q_i)_o = - D_i$$

resulting in Supplementary Eqs. (4)−(6).

### Calculations of the electronic structure in **1**

The geometry of the first coordination sphere of the Co^II^ in **1**, including only the donor oxygen atoms, is *D*_4*h*_. It represents a tetragonally elongated octahedron with two axial Co–O bonds to two water molecules (2.199 Å) and four equatorial Co-O bonds (2.05 Å) to two acac ligands. The crystallographic symmetry is *C*_2*h*_ (Supplementary Fig. 14). For spin-Hamiltonian parameters from ab initio NEVPT2 calculations, SOC, along with quasi-degenerate perturbation theory accounted for using all 10 *S* = 3/2 and 40 *S* = 1/2 non-relativistic states (roots) of the d^7^ Co^II^ configuration, was used to compute the ground and excited magnetic sublevels and to access the parameters of the spin-Hamiltonian in Eq. (4).

The ground ^4^*A*_g_ state splits into two sublevels, 169.8 cm^−1^ apart from each other, which in the approximation of an axial system would yield *D* = 84.9 cm^−1^. Diagonalization of the ZFS and the *g*-tensor yields eigenvalues and eigenvectors listed in Supplementary Table 8.

### VASP calculations of phonons

VASP^54^ calculations on **1**, **1-*d***_**4**_ and **1-*d***_**18**_ were conducted. Geometry optimizations were performed on the single-crystal X-ray structure of **1** at 100 K. The optimized structure completed at 0 T was used for the phonon calculations. Spin-polarized, periodic DFT calculations were performed using VASP with the Projector Augmented Wave^55,56^ method and the local density approximation (GGA)^57^ + *U* (*U* = 5.37)^55,58^ exchange correlation functional. An energy cut off was 900 eV for the plane-wave basis of the valence electrons. Total energy tolerance for electronic structure minimization was 10^−8^ eV. The optB86b-vdW, a non-local correlation functional that approximately accounts for dispersion interactions, was applied^59^. For the structure relaxation, a 1 × 3 × 1 Monkhorst–Pack mesh was applied. Phonopy^60^, an open source phonon analyzer, was used to create a 140 atom, 1 × 2 × 1 supercell structure. VASP was then employed to calculate the force constants on the supercell in real space using DFT. The crystal structure of **1** has *C*_*2h*_ symmetry. Jmol was used to create the Supplementary Movies. Since Raman and far-IR properties of **1** and **1-*d***_**4**_ near 115 cm^−1^ are similar, only the calculated phonons of **1-*d***_**4**_ are presented.

### Code availability

Electronic calculations were conducted with the ORCA code (https://orcaforum.cec.mpg.de/) which is free for academic use but commercial for industrial use. VASP (Vienna ab initio simulation package) for the periodic DFT phonon calculations is available at https://www.vasp.at/

### Data availability

The crystallographic coordinates for the structures of **1** at 100 K from single-crystal X-ray diffraction and **1-*d***_**18**_ at 4 K from powder neutron diffraction reported in this study have been deposited at the Cambridge Crystallographic Data Centre (CCDC), under deposition numbers CCDC 1842364 and CCDC 1842460, respectively. These data can be obtained free of charge from the Cambridge Crystallographic Data Centre via www.ccdc.cam.ac.uk/data_request/cif.

## Electronic supplementary material

Supplementary Information

Description of Additional Supplementary Files

Supplementary Movie 1

Supplementary Movie 2

Supplementary Movie 3

Supplementary Movie 4

Supplementary Movie 5
