# Supplementary material for: Spin–phonon couplings in transition metal complexes with slow magnetic relaxation
Source: Nat Commun. 2018 Jul 3;9:2572. doi: 10.1038/s41467-018-04896-0 (PMC6030095; doi:10.1038/s41467-018-04896-0)
Supplement: Supplementary file 1 — Supplementary Information [file 41467_2018_4896_MOESM1_ESM.pdf]

Supplementary Information

Spin-phonon couplings in transition metal complexes with slow magnetic relaxation

Duncan H. Moseley *et al.*

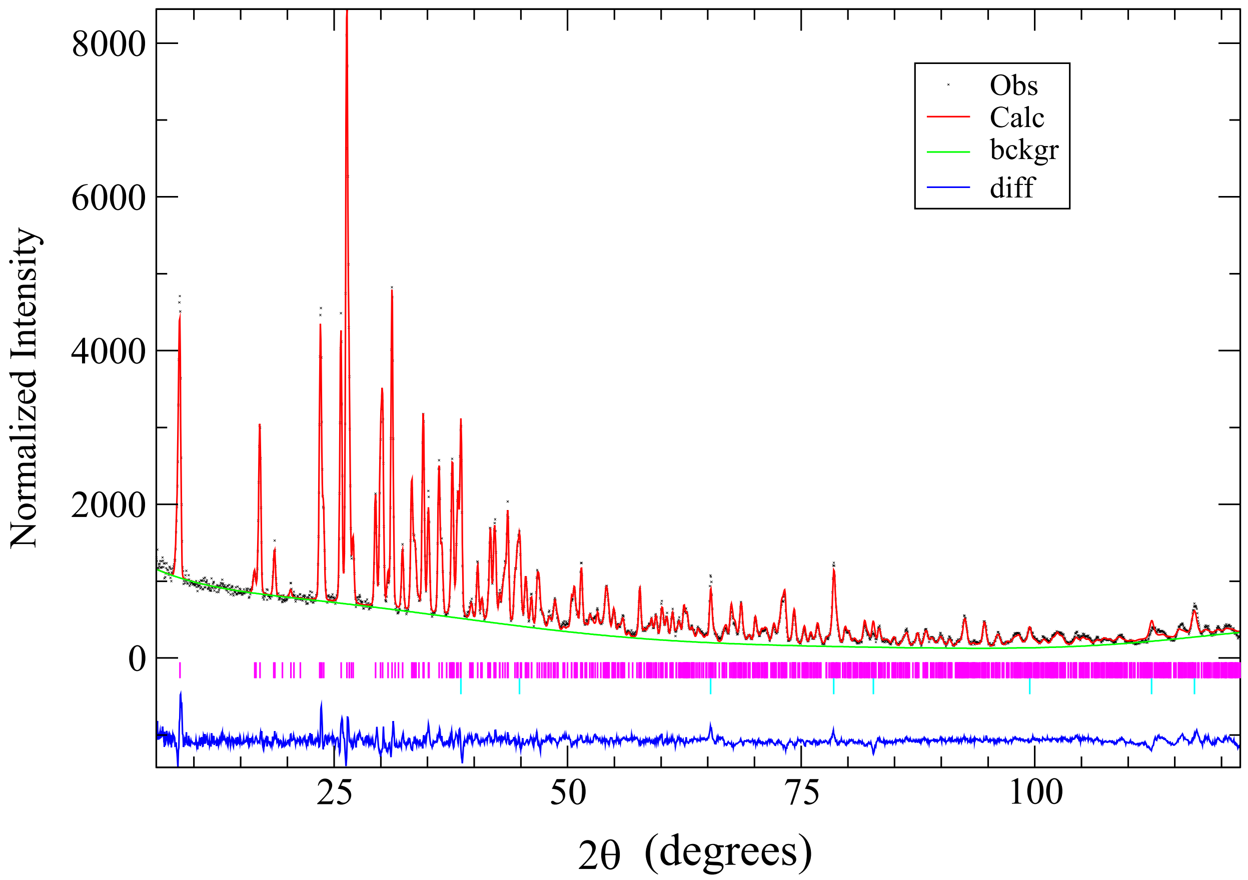

Supplementary Fig. 1. Rietveld refinement of neutron powder diffraction data. Data represent Co(acac-d_7)$_2$(D_2O)$_2$ (**1-*d*$_{18}$**) measured at 4 K at 0 T. Green lines, black crosses and red lines represent the background, experimental and calculated diffraction patterns, respectively. The blue line represents the difference between experimental and calculated patterns, with allowed reflections as pink tick marks and the aluminum sample holder as cyan ticks.

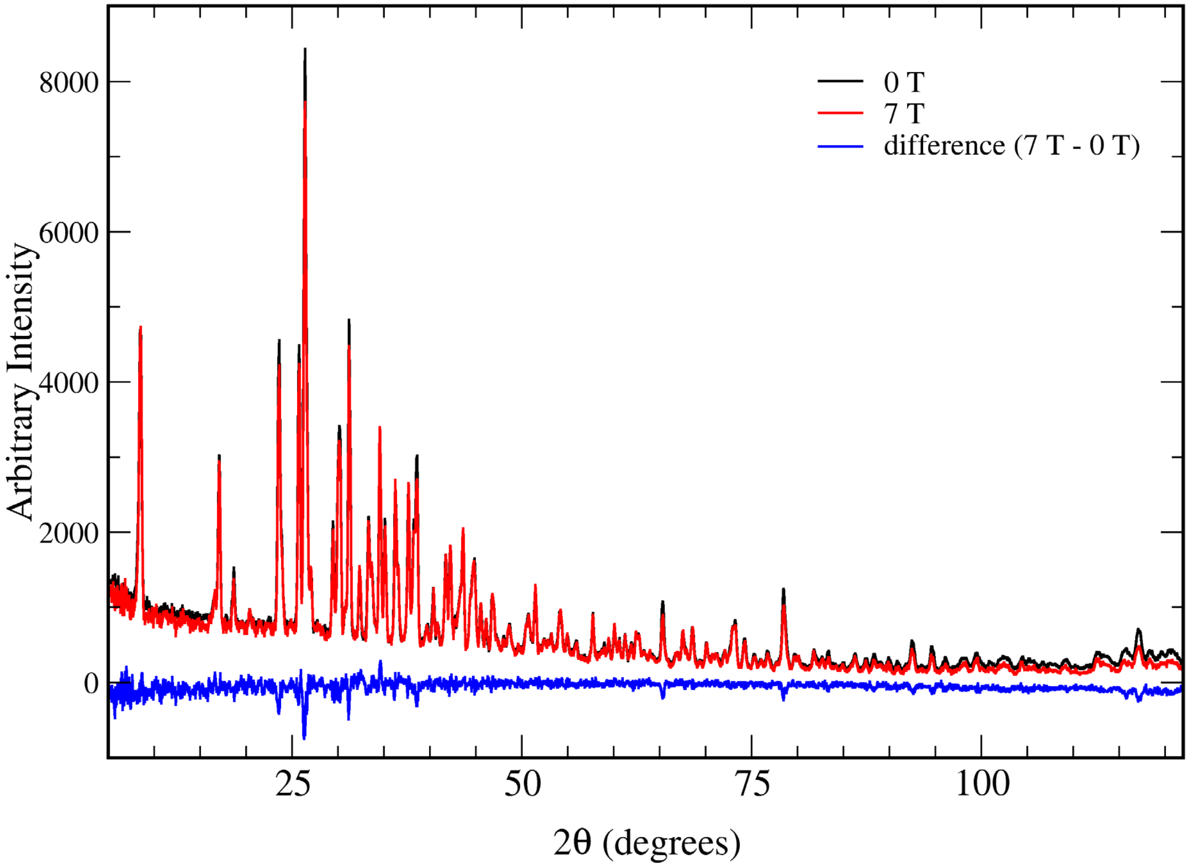

Supplementary Fig. 2. Neutron powder diffraction data. Data represent Co(acac-d_7)$_2$(D_2O)$_2$ (**1-d_{18}**) measured at 4 K and 0 T (black line) and 7 T (red line). The difference between the 0 and 7 T data (blue line) is also given. We note that, when the 7 T data were refined, there is little difference with the model at 0 T, given the restraints on the acac ligand. Single-crystal or high-resolution powder X-ray diffraction should be pursued in order to elucidate the differences, if any, between the 0 and 7 T structure.

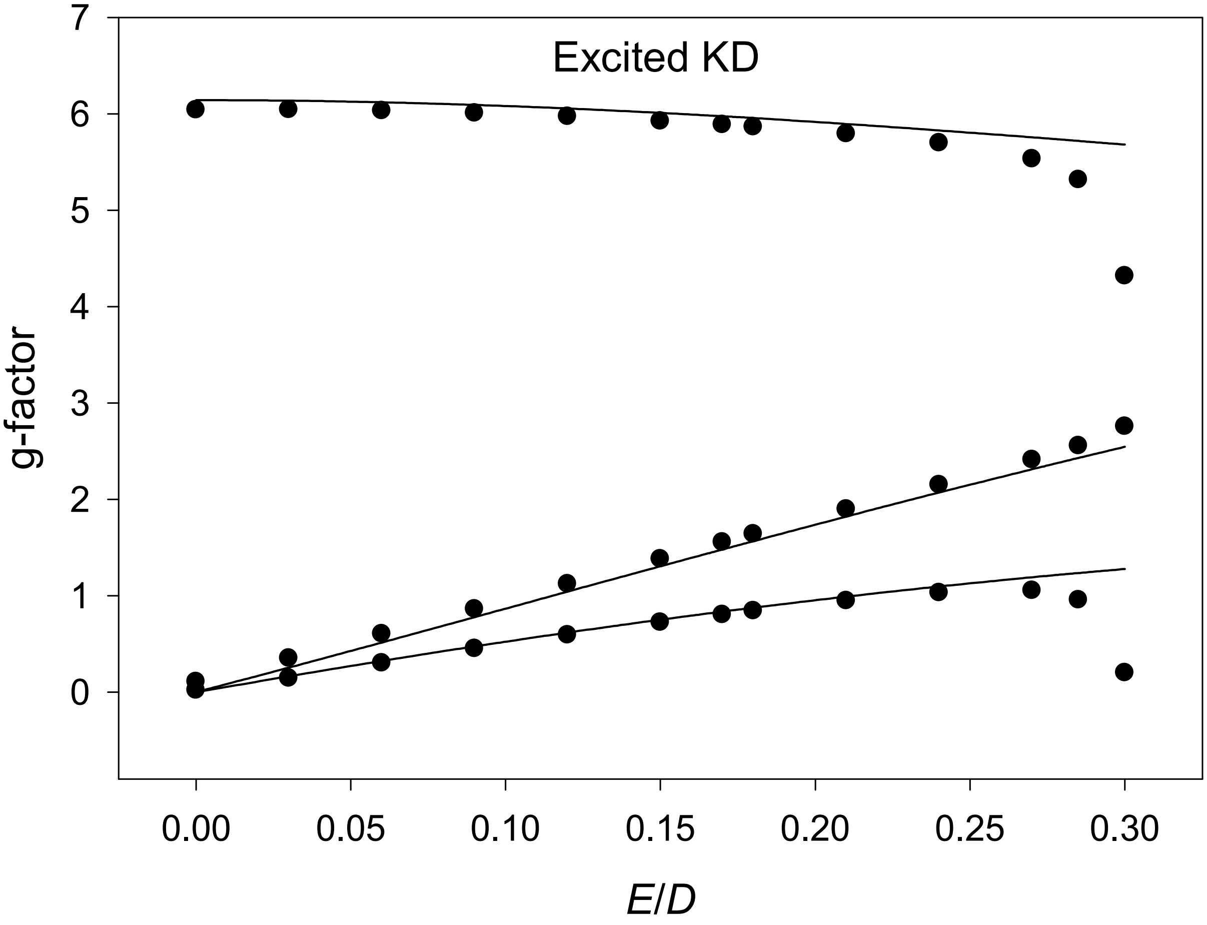

Supplementary Fig. 3. Effect of the vibronic couplings on the excited KD *g*-factors. Rhombograms at $B = 0.25$ T. Points are calculated using spin-phonon coupling. Lines are calculated using only spin.

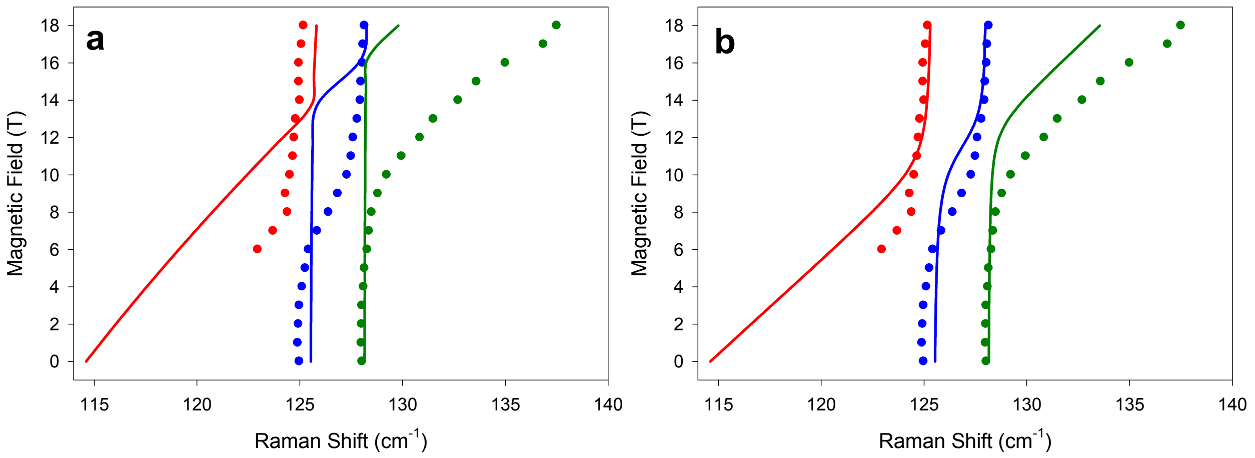

Supplementary Fig. 4. Fitting of Raman spectra. Simulated (solid lines) and experimental (circles) positions of field-dependent Raman spectra of **1** computed using the vibronic coupling model in Supplementary Eqs. (4-6) along with parameters specified in the Fig. 5 caption (main text) and field directions with respect to magnetic canonical axes: **a** $B||x$; **b** $B||y$.

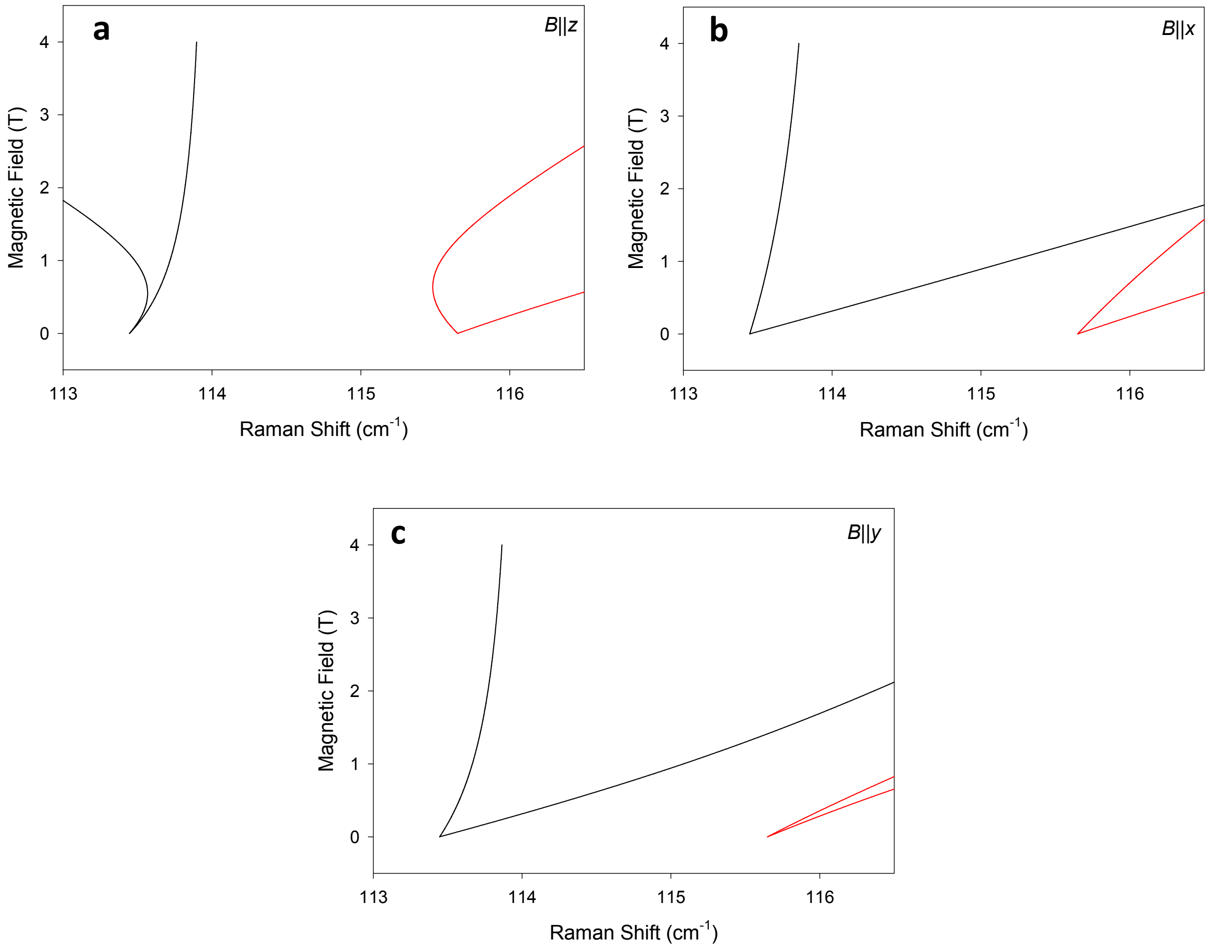

Supplementary Fig. 5. Magnetic and phonon computed positions. A model of two nearly degenerate states (lower state from a phonon at energy $\hbar\omega$ = 114 cm^{-1}; Upper state from a magnetic excitation at energy 115 cm^{-1}) that are coupled at 0 T. Parameters employed in the plots are: D = 55.2 cm^{-1}, E/D = 0.17, E_1 = 1 cm^{-1}, D_1 = 0.

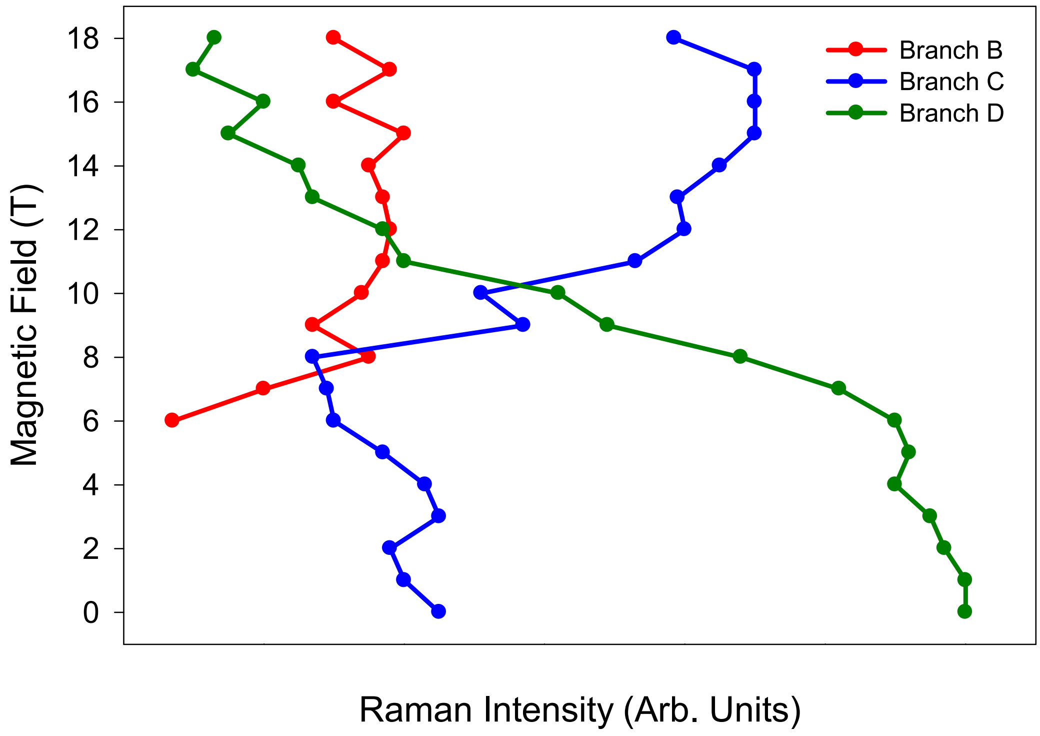

Supplementary Fig. 6. Intramolecular intensity distribution. Field dependence of Raman intensity of **1** corresponding to branches B, C and D. Irregularities in the reported features are due to noise.

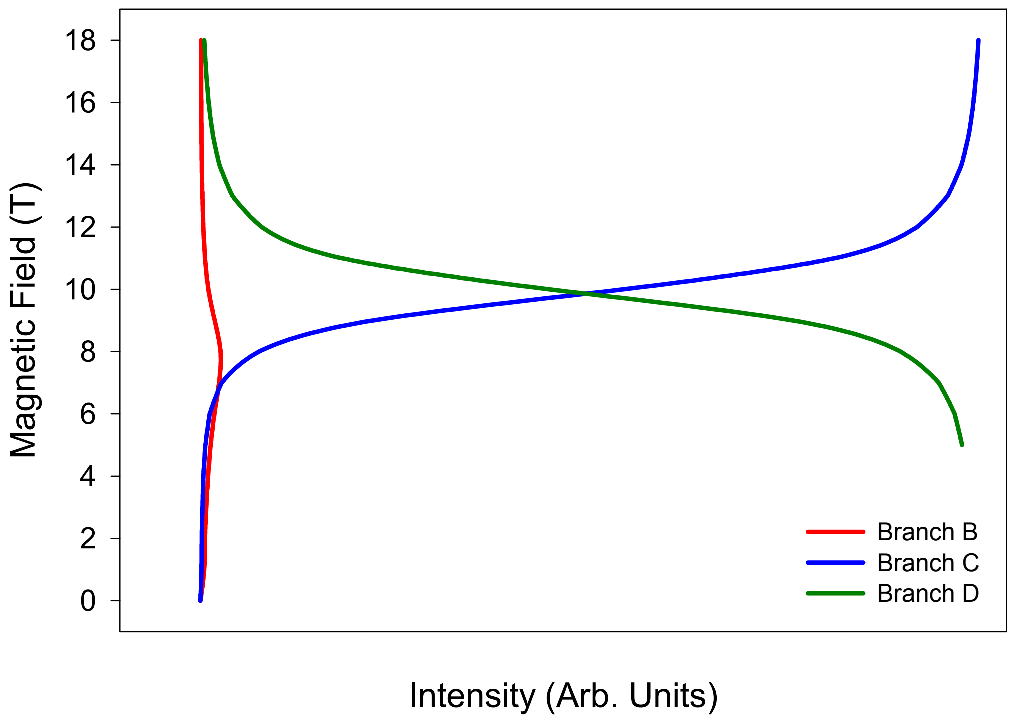

Supplementary Fig. 7. Computed intramolecular intensity distribution. Vibrational-intensity distribution of the Raman profile for branches B, C and D, computed using the spin-phonon coupling model with parameters specified in the Fig. 5 caption (main text) and the approximation given by Supplementary Eq. (7).

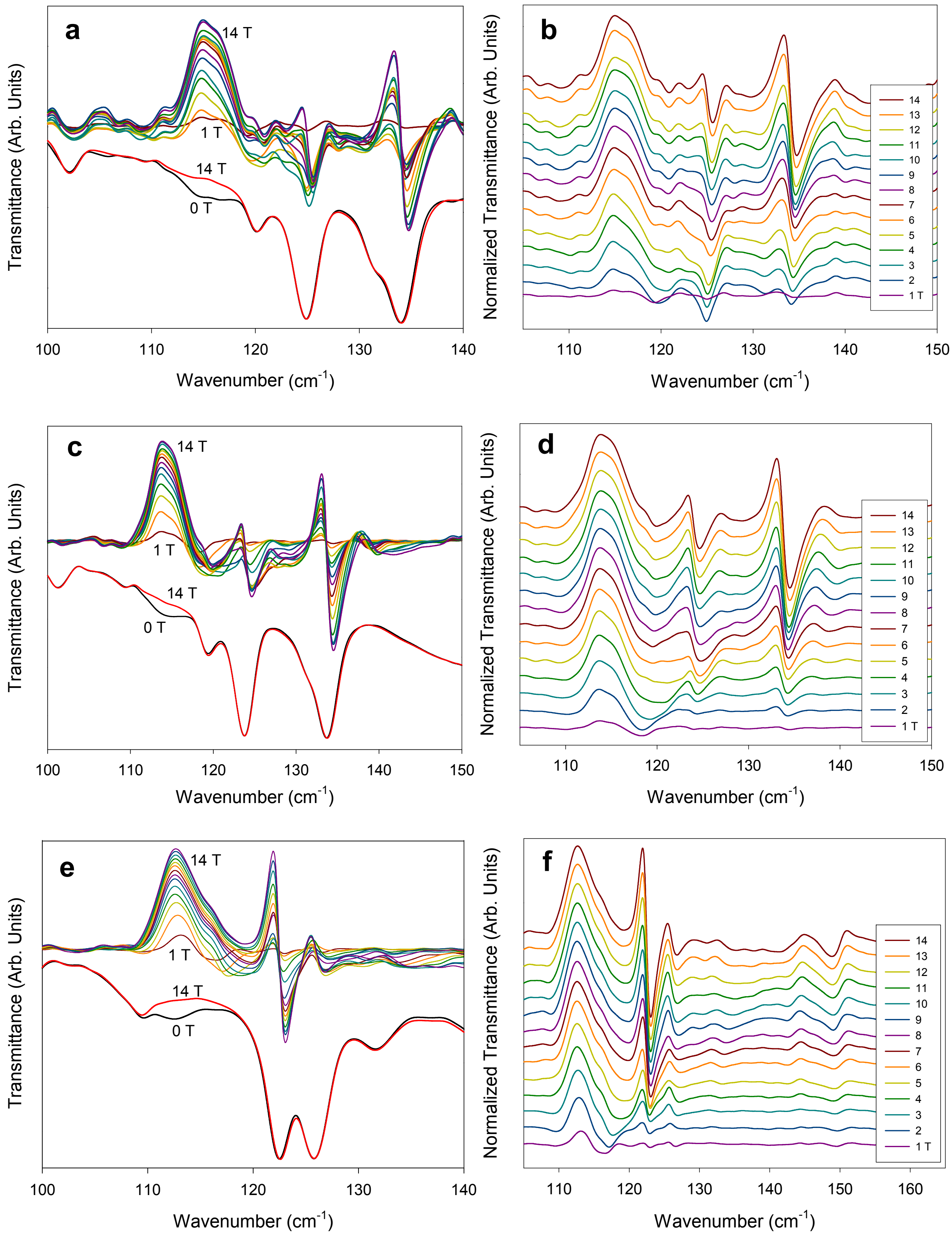
a
14 T
1 T
14 T
0 T
Transmittance (Arb. Units)
100
110
120
130
140
Wavenumber (cm^{-1})
b
Normalized Transmittance (Arb. Units)
14
13
12
11
10
9
8
7
6
5
4
3
2
1 T
110
120
130
140
150
Wavenumber (cm^{-1})
c
14 T
1 T
14 T
0 T
Transmittance (Arb. Units)
100
110
120
130
140
150
Wavenumber (cm^{-1})
d
Normalized Transmittance (Arb. Units)
14
13
12
11
10
9
8
7
6
5
4
3
2
1 T
110
120
130
140
150
Wavenumber (cm^{-1})
e
14 T
1 T
14 T
0 T
Transmittance (Arb. Units)
100
110
120
130
140
Wavenumber (cm^{-1})
f
Normalized Transmittance (Arb. Units)
14
13
12
11
10
9
8
7
6
5
4
3
2
1 T
110
120
130
140
150
160
Wavenumber (cm^{-1})

Supplementary Fig. 8. Far-IR transmittance spectra. **a** Far-IR transmittance (Bottom) and normalized (by the 0 T-Top) transmittance spectra of a powder sample of **1**. **b** Waterfall plots of the normalized spectra of **1**. **c** Far-IR transmittance (Bottom) and normalized (by the 0 T-Top) transmittance spectra of a powder sample of **1-d_4**. **d** Waterfall plots of the normalized spectra of **1-d_4**. **e** Far-IR transmittance (Bottom) and normalized (by the 0 T-Top) transmittance spectra of a powder sample of **1-d_{18}**. **f** Waterfall plots of the normalized spectra of **1-d_{18}**. The small feature at ~110 cm^{-1} is an artifact due to the eicosane matrix.

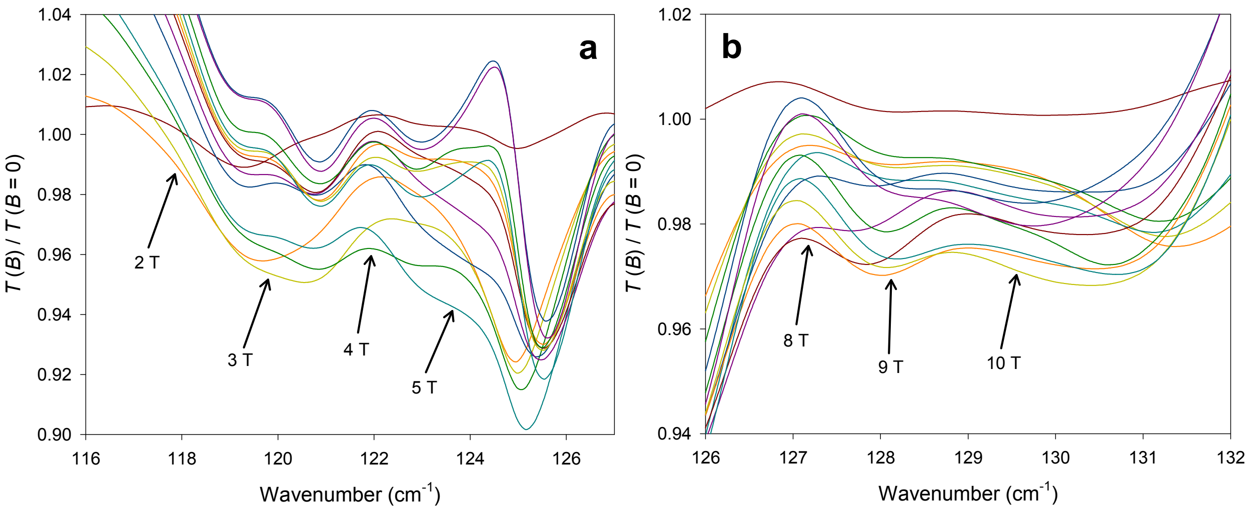

Supplementary Fig. 9. Expanded far-IR spectra of **1**. Normalized (by 0 T) transmittance far-IR spectra, illustrating the movement of the ZFS/magnetic peak. Decreased transmittance at different fields indicates an underlying intensity from the ZFS peak moving through the region.

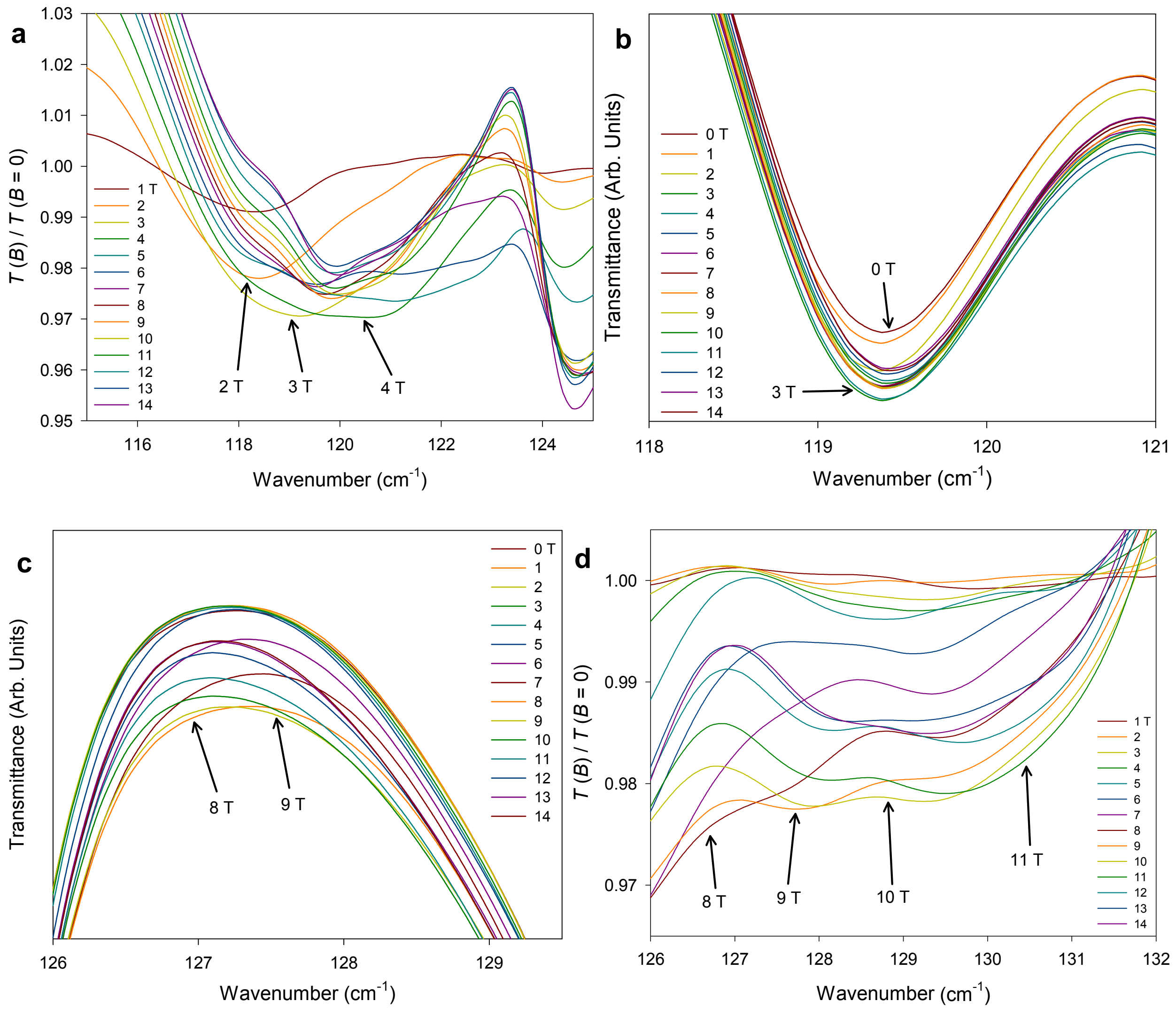

Supplementary Fig. 10. Expanded far-IR spectra of **1-*d*4**. **a**,**d** Normalized (by 0 T) transmittance; **b**,**c** Transmittance, illustrating the movement of the ZFS/magnetic peak. Decreased transmittance at different fields indicates an underlying intensity from the ZFS peak moving through the region.

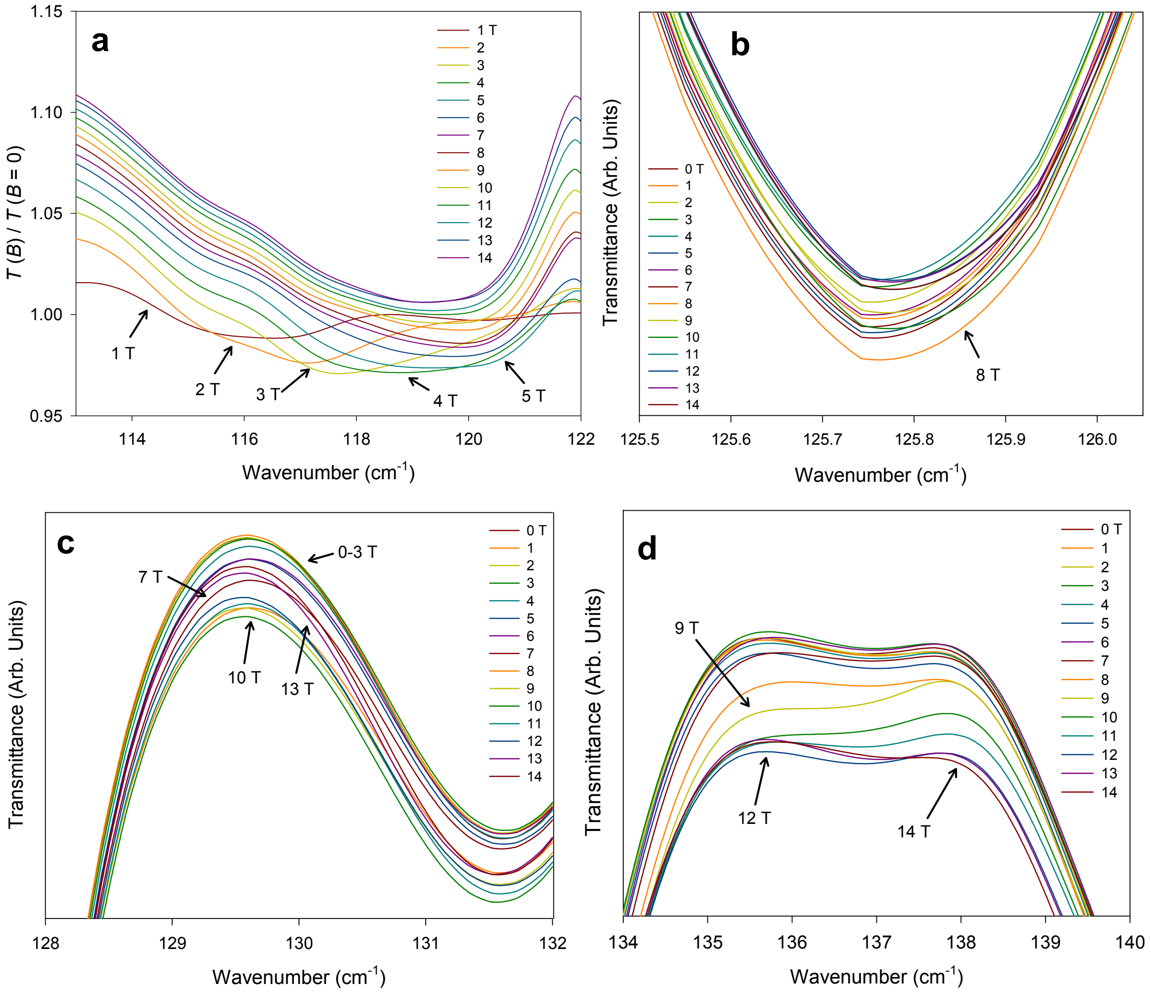

Supplementary Fig. 11. Expanded far-IR spectra of **1-d_{18}**. **a** Normalized (by 0 T) transmittance; **b-d** Transmittance, illustrating the movement of the ZFS peak. Decreased transmittance at different fields indicates an underlying intensity from the ZFS peak moving through the region.

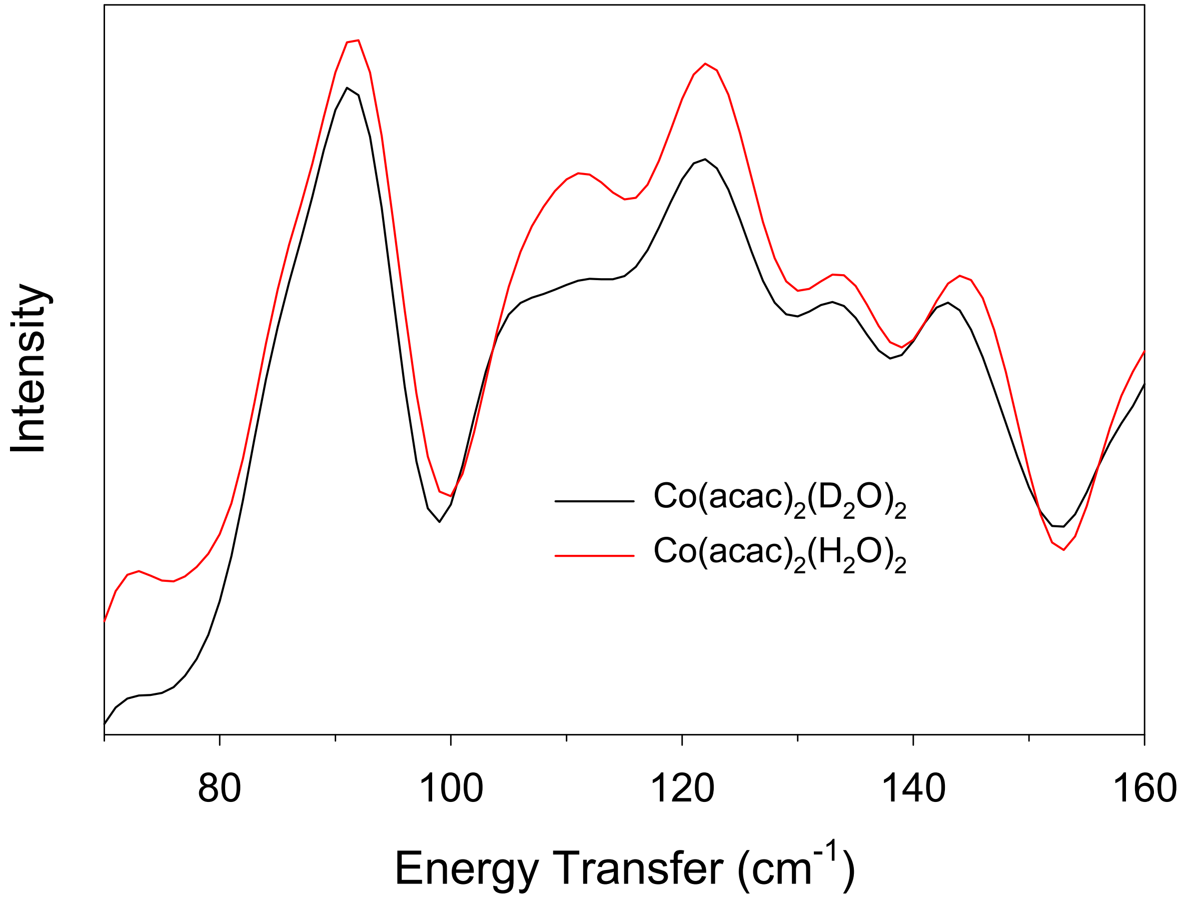

Supplementary Fig. 12. Comparison of the calculated phonon spectra of **1** and **1-*d*4**. In the energy region around 100 cm^{-1}, the modes are similar. Therefore, only the calculated modes of **1-*d*4** are discussed in the text.

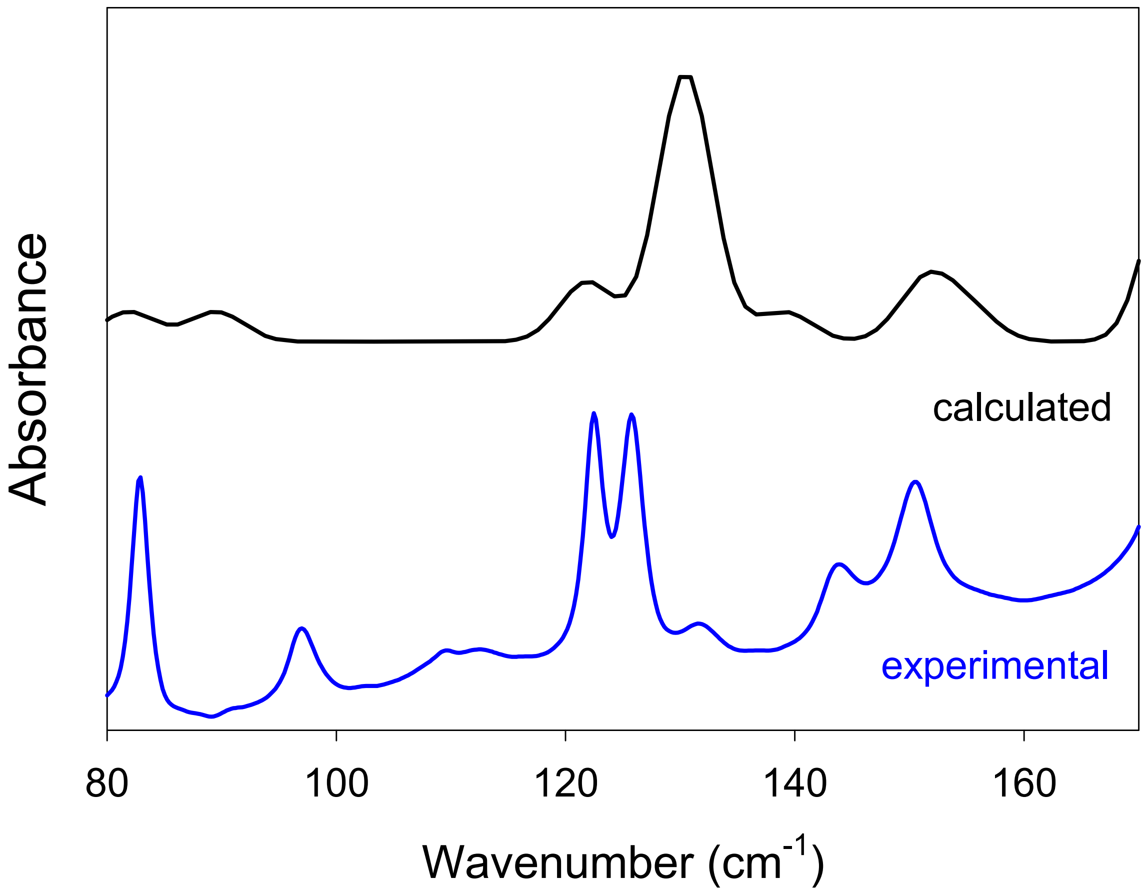

Supplementary Fig. 13. Comparison of far-IR data (0 T) and far-IR calculations in **1-*d*$_{18}$**.

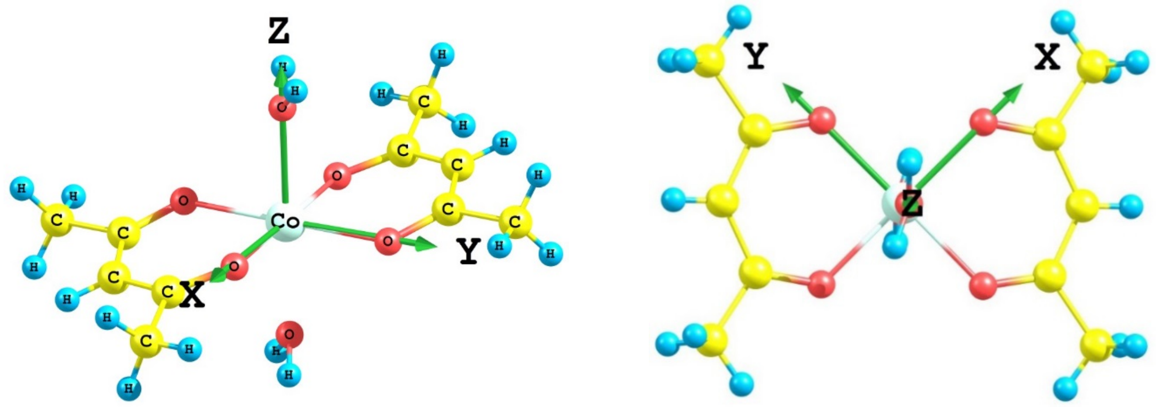

Supplementary Fig. 14. Geometric structure and choice of coordinate axes for **1**.

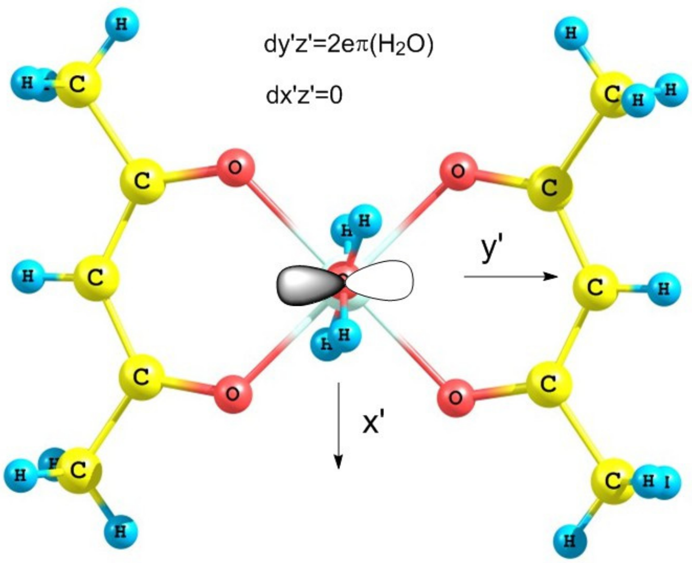

Supplementary Fig. 15. *π*-Anisotropy of the Co-OH2 bond.

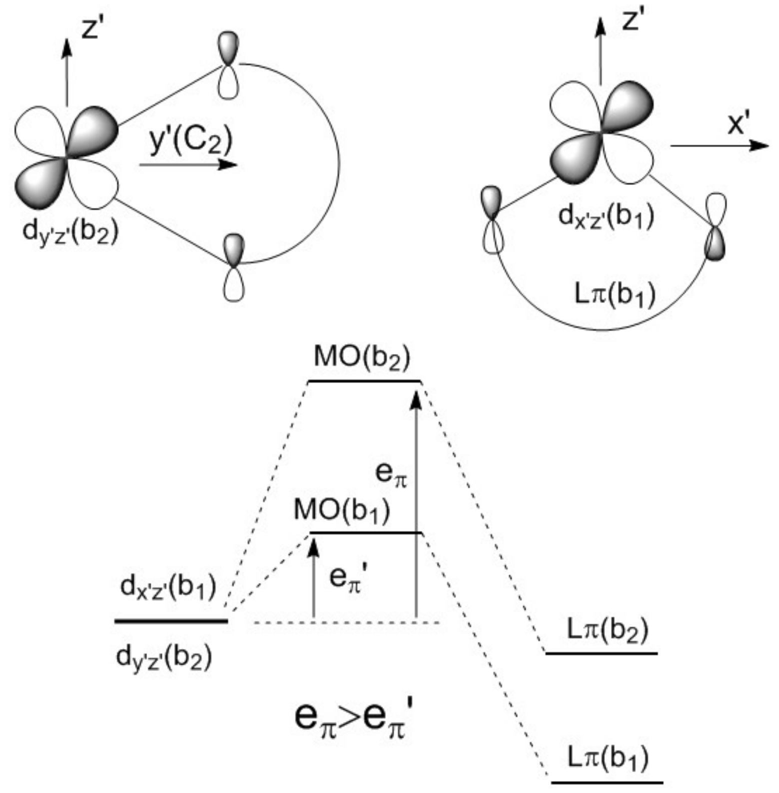

Supplementary Fig. 16. π-Anisotropic Co-acac interactions leading to a splitting of the $d_{x'z'}$, $d_{y'z'}$ orbital pair.

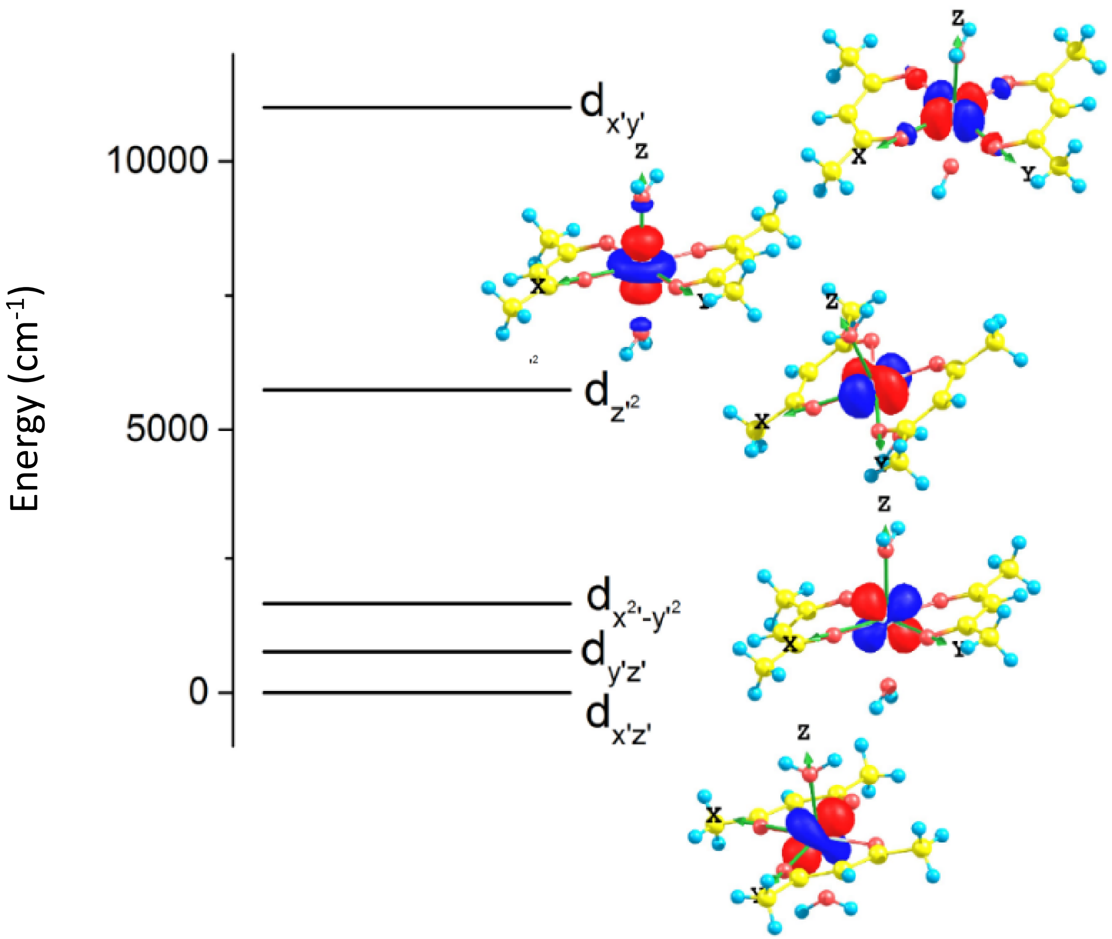

Supplementary Fig. 17. *Ab initio* (NEVPT2) ligand-field 3d-orbital energies of **1**.

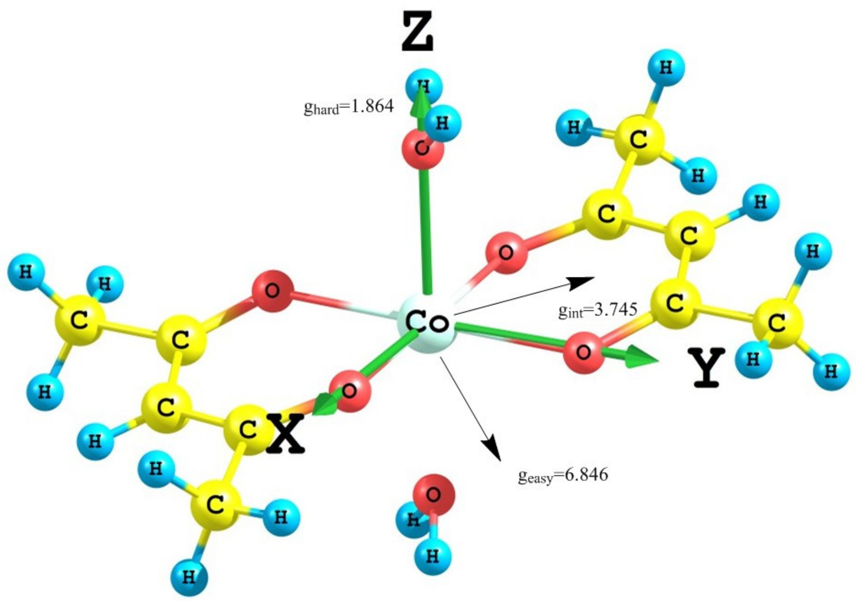

Supplementary Fig. 18. Effective, principle *g*-tensor components. For ground-state KD_1 and their orientation with respect to the molecular frame from NEVPT2/SOC-state interaction calculations of **1**, the smallest *g*-tensor value is collinear with the molecular *z*-axis. The *g*-tensor within the easy equatorial plane is split into a large value of 6.846 contenting an angle of only 10° with the bisector of the inter-chelate O-Co-O angle. The intermediate principle *g*-value contends an angle of 10° with the direction bisecting each O-Co-O chelate angle.

Supplementary Table 1. Neutron powder diffraction Rietveld refinement results.

Data represents **1-*d*$_{18}$** measured at 4 K. Goodness-of-fit parameters: χ^2 = 2.98, Rp = 5.27%, wRp = 7.53% and wR_{exp} = 4.36%. Space group $P2_1/c$; λ = 1.5397 Å; Unit cell parameters: *a* = 10.7672(5) Å, *b* = 5.3425(2) Å, *c* = 11.0569(5) Å, *β* = 105.782(4)° and *V* = 612.06(5) Å^3.

**Name**	***x***	***y***	***z***	***B*$_{iso}$ (Å^2)**	**Occupancy**
Co1	0.5	0.5	0.5	4.38(15)	1
O1	0.3513(12)	0.2768 (18)	0.4043(11)	4.38(15)	1
O2	0.3805(11)	0.660(2)	0.5872(10)	4.38(15)	1
O3	0.4361(11)	0.783(2)	0.3582(12)	4.38(15)	1
C1	0.1837(10)	0.5135(18)	0.4548(8)	3.59(8)	1
C2	0.2324(9)	0.3279(19)	0.3905(8)	3.59(8)	1
C3	0.1440(10)	0.1665(18)	0.3027(8)	3.59(8)	1
C4	0.2603(8)	0.6776(17)	0.5492(9)	3.59(8)	1
C5	0.1888(9)	0.8612(17)	0.6017(9)	3.59(8)	1
D1	0.0851(10)	0.5363(18)	0.4275(9)	5.03(8)	1
D3C	0.0418(10)	0.2226(17)	0.2825(9)	5.03(8)	1
D3D	0.1642(10)	0.1523(18)	0.2099(9)	5.03(8)	1
D3E	0.1472(10)	-0.0302(19)	0.3332(9)	5.03(8)	1
D5A	0.0864(9)	0.861(2)	0.5649(9)	5.03(8)	1
D5B	0.2090(9)	0.837(2)	0.7022(10)	5.03(8)	1
D5C	0.2269(10)	1.0482(18)	0.5923(8)	5.03(8)	1
D3A	0.4385(11)	0.7761(16)	0.2743(10)	5.03(8)	1
D3B	0.4182(10)	0.954(2)	0.3750(9)	5.03(8)	1

Supplementary Table 2. Band maxima positions in Raman spectra.

**Branch (cm^{-1})**	**A**	**B**	**C**	**D**	**E**
**1**					
$B = 0$	116.29	-	124.98	128.03	138.90
$B = 6$	116.65	122.95	125.43	128.28	139.17
$B = 7$	116.88	123.70	125.85	128.37	139.08
$B = 18$	118.94	125.18	128.15	137.50	141.25
**1-*d*$_4$**					
$B = 0$	115.46	-	124.11	128.41	138.32
$B = 5$	116.08	122.35	124.57	128.47	138.47
$B = 6$	116.27	122.92	125.07	128.73	138.64
$B = 14$	118.57	124.32	128.27	137.47	140.47
**1-*d*$_{18}$**					
$B = 0$	112.42	116.80	-	-	-
$B = 4.5$	113.60	119.70	-	-	-
$B = 14.0$	114.10	-	-	-	-

Supplementary Table 3. Comparisons of peak positions of phonons near the ZFS peak. All units are given in cm^{-1}.

1-d_4; Calculated energy	Symmetry[a]	Peak label	Raman	Far-IR
99.1	A_u $(\Gamma^-_{3,4})$	-	-	101.2
103.6	A_g $(\Gamma^+_{3,4})$	-	96.8	-
107.0	B_g $(\Gamma^+_{3,4})$	-	100.2	-
109.2	A_g $(\Gamma^+_{3,4})$	A	115.2	-
120.3	A_u $(\Gamma^-_{3,4})$	-	-	120.0
125.0	B_u $(\Gamma^-_{3,4})$	-	-	123.4
126.0	B_g $(\Gamma^+_{3,4})$	C	124.4	-
129.3	A_g $(\Gamma^+_{3,4})$	D	129.0	-
135.6	B_u $(\Gamma^-_{3,4})$	-	-	131.9
140.1	A_u $(\Gamma^-_{3,4})$	-	-	133.8
142.7	B_g $(\Gamma^+_{3,4})$	E	139.2	-

1-d_{18}; Calculated energy	Symmetry[a]	Peak label	Raman	Far-IR
91.3	B_g $(\Gamma^+_{3,4})$	-	90.9	-
93.5	A_u $(\Gamma^-_{3,4})$	-	-	96.9
95.7	A_g $(\Gamma^+_{3,4})$	-	96.4	-
103.3	A_g $(\Gamma^+_{3,4})$	-	106.2[b]	-
109.8	A_g $(\Gamma^+_{3,4})$	-	106.2[b]	-
114.3	A_u $(\Gamma^-_{3,4})$	-	-	weak
116.3	B_g $(\Gamma^+_{3,4})$	A	112.5 (0 T)<br>115 (6 T)	-

116.6	B_u ($\Gamma^{-}_{3,4}$)	-	-	weak

[a] Symmetries of vibrations are listed following Mulliken notations for the C_{2h} group. The vibronic states (in parentheses) are denoted using notations for the C_{2h}' double group as defined in Table 15 of ref. 44 (in the main text).

[b] Either one of those calculated modes could be attributed to the experimental phonon observed at 106.2 cm^{-1}. However, without information on the calculated intensity of these Raman-active peaks, a definitive assignment cannot be made. While there are two calculated peaks in this region, only the one with experimental intensity is observed.

Supplementary Table 4. Vibronic model parameters.

Parameters and root-mean-square (RMS) deviations (cm^{-1}) from a best fit to field-dependent Raman spectra of **1** ($B||z$) with variable E/D values. E/D value is fixed in each fit. (The value of the energy gap $2D' = (D^2 + 3E^2)^{1/2} = 115$ cm^{-1} is taken from the field-dependent far-IR data.)

E*/*D	**0.0**	**0.05**	**0.10**	**0.15**	**0.17**	**0.20**	**0.25**	**0.30**
g	1.413	1.602	1.535	1.467	1.492	1.499	1.558	1.648
$\hbar\omega_1$	125.4	125.2	125.3	125.4	125.4	125.4	125.4	125.4
$\hbar\omega_2$	128.1	128.0	128.0	128.1	128.1	128.1	128.0	128.0
$\hbar\omega_3$	141.3	144.1	143.5	140.0	139.5	138.8	138.0	137.5
E_1	1.13	1.14	1.15	1.08	1.14	1.25	1.17	0.89
E_2	0.94	0.94	0.95	1.15	0.88	1.25	1.07	0.84
E_3	2.58	5.97	4.81	2.56	2.66	2.44	2.47	2.74
RMS dev	0.581	0.579	0.581	0.596	0.595	0.613	0.630	0.674

Supplementary Table 5. Avoided crossing parameters.

Experimental and computed energy (cm^{-1}) and B (Tesla) of avoided crossings (x) and ΔE (cm^{-1})/ΔB (Tesla) slopes of the shifting magnetic peaks for **1**, **1-*d*$_4$** and **1-*d*$_{18}$**.

	x_1		x_2		x_3		
	**Energy (cm^{-1})**	***B* (T)**	**Energy**	***B***	**Energy**	***B***	**ΔEnergy (cm^{-1})/Δ*B* (T)**
Exp. with **1**	125.05	7.638	127.99	9.432	138.71	17.541	1.362
Exp. with **1-*d*$_4$**	124.25	6.488	128.54	8.612	-	-	2.017
Exp. with **1-*d*$_{18}$**	114.61	0	-	-	-	-	-
Computed $B \| x$	125.12	11.49	129.10	14.48	-	-	1.331
Computed $B \| y$	125.23	8.03	129.31	11.04	-	-	1.356
Computed $B \| z$	125.46	6.04	129.48	8.01	139.08	12.93	1.972

Supplementary Table 6. Distortion of the O-Co-O bond angles in the equatorial plane. Compared with the spin-phonon coupling constants $|\mathit{\Lambda}|$.

Complex	Calculated vibration (cm^{-1}) (Peak label)	Equilibrium bond angle (°)	Distorted bond angle (°)	Change in bond angle (°)	Spin-phonon coupling constants $|\mathit{\Lambda}|$
**1-*d*₄**	126.0 (C)	90.75	90.93	-0.18	0.95(15)
**1-*d*₄**	129.3 (D)	90.75	90.85	-0.10	1.00(10)
**1-*d*₄**	142.7 (E)	90.75	92.15	-1.4	2.05(10)
**1-*d*₁₈**	116.3 (A)	90.75	89.88	0.87	2.15(10)

Supplementary Table 7. Electronic state energies.

Non-relativistic $S = 3/2$ states and their splitting on symmetry lowering from O_h to D_{4h} and D_{2h} (NEVPT2 results). Here we have added a new column with notations of the non-relativistic terms according to the C_{2h} point group.

O_h	D_{4h}	D_{2h}	C_{2h}	Energy (cm^{-1})
$^4T_{1g}$	$^4A_{2g}$<br>^4E_g	$^4B_{1g}$<br>$^4B_{2g}$, $^4B_{3g}$	^4A_g<br>^4B_g, ^4B_g	0<br>707, 1175
$^4T_{2g}$	$^4B_{2g}$<br>^4E_g	^4A_g<br>$^4B_{2g}$, $^4B_{3g}$	^4A_g<br>^4B_g, ^4B_g	6352<br>9152, 9362
$^4A_{2g}$	$^4B_{1g}$	$^4B_{1g}$	^4A_g	17548
$^4T_{1g}$	^4E_g<br>$^4A_{2g}$	$^4B_{2g}$, $^4B_{3g}$<br>$^4B_{1g}$	^4B_g, ^4B_g<br>^4A_g	20806, 21583<br>26363

Supplementary Table 8. Parameters of the spin-Hamiltonian.[a]

	D_{xx}	D_{yy}	D_{zz}	g_x	g_y	g_z
	-13.13	-41.12	54.26	2.574	2.846	1.889
x	0.78	0.58	0.24	0.79	0.58	0.19
y	-0.57	0.81	-0.12	-0.58	0.81	-0.08
z	-0.26	-0.04	0.96	-0.20	-0.05	0.98

[a] Deduced from NEVPT2 results and a mapping of the lowest four SOC eigenfunctions onto the spin-Hamiltonian in Eq. (4).

Supplementary Note 1. Crystal structure of 1-d_{18} by powder neutron diffraction

A neutron diffraction pattern (Supplementary Figs. 1-2) on a sample of **1-d_{18}** (in the aluminum sample holder) was measured at 4 K at the constant (λ = 1.5397 Å) wavelength from a Cu(311) monochrometer of the BT-1 neutron powder diffractometer at the National Institute for Standards and Technology (NIST) at 0 and 7 T. The 7 T data were collected to extract information about the crystal structure when the external field was applied. Rietveld refinement was performed using the TOPAS academic software suite[1]. The pattern shows only reflections expected from the nuclear components of the desired phase, with a second phase arising from the aluminum cell. The phase of Co(acac-d_7)$_2$(D_2O)$_2$ (**1-d_{18}**) was modeled after the structure of **1** from the single-crystal X-ray diffraction at 100 K (CCDC 1842364). The powder pattern is in excellent agreement, with the molecular structure allowed to relax with soft restraints for C-C bond lengths to 1.40 Å and 1.45 Å (tolerance 0.05, weight 1), a flat geometry restraint on the acac-d_7 ligand, and all atoms of the same type constrained to have equivalent B_{iso} parameters. Due to the use of neutron scattering, the D atoms were unambiguously located for the structure, and their locations allowed to refine freely, along with the O locations. Attempts were made to refine both H and D occupations on the acac-d_7 and D_2O ligand fragments but no improvement in the fits were obtained, with D occupations refining close to unity. Hence, the occupations were fixed at this nominal value. Results of the refinement are given in the cif file (CCDC 1842460) and summarized in Supplementary Table 1.

Hydrogen bonding interactions are identified between the aqua groups and equatorial O atoms making up the coordination sphere of the Co^{II} ion. Distance are 2.021(15) Å and 1.929(15) Å for D3A-O2 and D3B-O1, respectively. Interactions over longer distances are evident between

D5B and D5C to O2, and D3D and D3E to O1 of the order of 2.6 Å to 2.7 Å, that are likely responsible for the methyl group orientations in the solid.

Note: Certain commercial equipment, instruments, or materials are identified in this document. Such identification does not imply recommendation or endorsement by the National Institute of Standards and Technology nor does it imply that the products identified are necessarily the best available for the purpose.

Supplementary Note 2. Alternative equation for the analysis of spin-phonon couplings in Raman spectra

Eq. (2) describing the coupling between magnetic $|\varphi_{sp}\rangle$ and phonon $|\varphi_{ph}\rangle$ peaks in the Raman spectra in Fig. 2 (main text) may also be expressed in Supplementary Eq. (1).

$$E_{\pm} = \frac{E_{sp} + E_{ph}}{2} \pm \sqrt{\left(\frac{E_{sp} - E_{ph}}{2}\right)^2 + \Lambda^2} \quad (1)$$

Supplementary Note 3. Use of the vibronic model for the magnetic field dependent Raman spectra of 1

Taking a representation of the five basis functions $|M_S,n_1,n_2,n_3\rangle$, M_S = 1/2,3/2; n_i = 0,1; i = 1,2,3 as a simple product $|M_S,n_1,n_2,n_3\rangle = |M_S\rangle|n_1\rangle|n_2\rangle|n_3\rangle$, the following Hamiltonian matrix of the problem results: $\left|-1/2,0,0,0\right\rangle \rightarrow \left|3/2,0,0,0\right\rangle, \left|-1/2,1,0,0\right\rangle, \left|-1/2,0,1,0\right\rangle, \left|-1/2,0,0,1\right\rangle$

$$\begin{bmatrix} 2D+2\beta_B g_z B_z & E_1 & E_2 & E_3 \\ E_1 & \hbar\omega_1 & 0 & 0 \\ E_2 & 0 & \hbar\omega_2 & 0 \\ E_3 & 0 & 0 & \hbar\omega_3 \end{bmatrix} \qquad (2)$$

At zero field, the energy gap $2D$ ($|\pm3/2,0,0,0\rangle - |\mp1/2,0,0,0\rangle$) is 115 cm^{-1} and thus below the energy of the three single excited vibrations at $\hbar\omega_1$ = 125, $\hbar\omega_2$ = 128 and $\hbar\omega_3$ = 139 cm^{-1}. However, this gap is tunable by the field and turns to zero at fields B obeying the resonance condition:

$$2D+2\beta_B g_z B_z = \hbar\omega_i \qquad (3)$$

From this and taking the best-fit value g_z = effective g = 1.46, one can easily rationalize the shape of the experimental data points in Fig. 4. Particularly the increase of the values of the field at the avoided crossing points x_1, x_2 and x_3 is well reproduced. Focusing on peak B of the spin-phonon plot and passing through the avoided crossing point x_1, magnetic excitation $\left|-1/2,0,0,0\right\rangle \rightarrow \left|3/2,0,0,0\right\rangle$ converts to a vibrational excitation $\left|-1/2,0,0,0\right\rangle \rightarrow \left|-1/2,1,0,0\right\rangle$.

There is twice the change in character of branch C where the low-field region due to

$\left|-1/2,0,0,0\right\rangle \rightarrow \left|-1/2,1,0,0\right\rangle$ is replaced by $\left|-1/2,0,0,0\right\rangle \rightarrow \left|3/2,0,0,0\right\rangle$ at x_1, while the latter in turn converts to $\left|-1/2,0,0,0\right\rangle \rightarrow \left|-1/2,0,1,0\right\rangle$ at x_2. In branch D, $\left|-1/2,0,0,0\right\rangle \rightarrow \left|-1/2,0,1,0\right\rangle$ is converted to $\left|-1/2,0,0,0\right\rangle \rightarrow \left|3/2,0,0,0\right\rangle$ at x_2, but the second change to $\left|-1/2,0,0,0\right\rangle \rightarrow \left|-1/2,0,0,1\right\rangle$, expected to appear at higher field, falls outside the range of field values. Finally, in branch E, initially the $\left|-1/2,0,0,0\right\rangle \rightarrow \left|-1/2,0,0,1\right\rangle$ excitation goes to $\left|-1/2,0,0,0\right\rangle \rightarrow \left|3/2,0,0,0\right\rangle$ at the highest field of 17.54 T (x_3). As stated above, in the approximation of a purely axial anisotropy, the $\left|-1/2, 0, 0, 0\right\rangle \rightarrow \left|3/2, 0, 0, 0\right\rangle$ excitation is formally magnetic-dipole-forbidden, but gains intensity via mixing of the $\left|-1/2, 0, 0, 0\right\rangle$ and $\left|3/2, 0, 0, 0\right\rangle$ states via the orthorhombic $E(\hat{S}_x^2 - \hat{S}_y^2)$ term. Another source of intensity for this transition is the mixing with the Raman-active phonon in the vicinity of an avoided crossing point and apparently this mixing dominates the Raman intensity of the entire spectrum. Thus, in peak B at a low field far from the avoided crossing point x_1, mixing between $\left|-1/2,0,0,0\right\rangle \rightarrow \left|3/2,0,0,0\right\rangle$ and $\left|-1/2,0,0,0\right\rangle \rightarrow \left|-1/2,1,0,0\right\rangle$ is small and readily explains the absence of experimental points. As stated above, the *E* term is large, such that a more precise consideration of the data in Fig. 4 (main text) is needed. In order to analyze this phenomenon of spin-phonon (vibration) entanglement in more quantitative terms, we have extended the model of Supplementary Eq. (2) to a Hamiltonian matrix taking into account all basis functions $|M_S,n_1,n_2,n_3\rangle$ (M_S = 3/2, 1/2, -1/2, -3/2; n_1, n_2, n_3 = 0, 0, 0; 1, 0, 0; 0, 1, 0; 0, 0, 1). This leads to the four-by-four block form of Supplementary Eq. (4):

$$\mathbf{H}_{\mathbf{eff}} = \begin{bmatrix} \mathbf{H}_{\mathbf{S}} & \mathbf{H}_{\mathbf{S-vib1}} & \mathbf{H}_{\mathbf{S-vib2}} & \mathbf{H}_{\mathbf{S-vib3}} \\ \mathbf{H}_{\mathbf{S-vib1}} & \mathbf{H}_{\mathbf{S}} + \hbar\omega_1\mathbf{I} & \mathbf{0} & \mathbf{0} \\ \mathbf{H}_{\mathbf{S-vib2}} & \mathbf{0} & \mathbf{H}_{\mathbf{S}} + \hbar\omega_2\mathbf{I} & \mathbf{0} \\ \mathbf{H}_{\mathbf{S-vib3}} & \mathbf{0} & \mathbf{0} & \mathbf{H}_{\mathbf{S}} + \hbar\omega_3\mathbf{I} \end{bmatrix} \tag{4}$$

The same expressions for the spin-Hamiltonian matrix $\mathbf{H}_{\mathbf{S}}$ in Supplementary Eq. (5) and the spin-phonon coupling sub-diagonal matrices $\mathbf{H}_{\mathbf{S-vib1}}$, $\mathbf{H}_{\mathbf{S-vib2}}$ and $\mathbf{H}_{\mathbf{S-vib3}}$ in Supplementary Eq. (6) apply, now referring to the coupling of the spin S with the three normal modes Q_1, Q_2 and Q_3.

$$\mathbf{H}_{\mathbf{S}} = \begin{bmatrix} D+\frac{3}{2}G_{zz} & \frac{\sqrt{3}}{2}(G_{xx}-iG_{yy}) & \sqrt{3}E & 0 \\ \frac{\sqrt{3}}{2}(G_{xx}+iG_{yy}) & -D+\frac{1}{2}G_{zz} & (G_{xx}-iG_{yy}) & \sqrt{3}E \\ \sqrt{3}E & (G_{xx}+iG_{yy}) & -D-\frac{1}{2}G_{zz} & \frac{\sqrt{3}}{2}(G_{xx}-iG_{yy}) \\ 0 & \sqrt{3}E & \frac{\sqrt{3}}{2}(G_{xx}+iG_{yy}) & D-\frac{3}{2}G_{zz} \end{bmatrix} \tag{5}$$

$$\mathbf{H}_{\mathbf{S-vib}} = \begin{bmatrix} D' & 0 & E' & 0 \\ 0 & -D' & 0 & E' \\ E' & 0 & -D' & 0 \\ 0 & E' & 0 & D' \end{bmatrix} \tag{6}$$

where $G_{ii} = \beta g_i B_i$ denotes the Zeeman interaction for fields in $i = x, y, z$ directions.

This Hamiltonian has been diagonalized under the following simplifying assumptions:

1) Parameters of the spin-Hamiltonian D, E, g_x, g_y and g_z are not reliably known. In the simulation of the Raman spectrum, a single isotropic g was used.

2) Field-dependent far-IR spectra of **1** allow us to set with confidence the energy separation ΔE (expressed as $2\sqrt{D^2+3E^2}$) between the ground-state KD_1 and excited-state KD_2 at 115 cm^{-1}.

3) We have chosen the *ab initio* (NEVPT2) ratio $\lambda = E/D = 0.17$ to derive $D = 55.16$ and $E = 9.38$ cm^{-1} but, as pointed out above, simulations of the experimental Raman data turn out to be insensitive to the E/D ratio (Supplementary Table 4).

4) The parameters D_1, D_2 and D_3 for the three vibrations were set to zero.

5) $\hbar\omega_1$, $\hbar\omega_2$ and $\hbar\omega_3$ and E_1, E_2 and E_3 have been treated as adjustable parameters and obtained from a best fit to the reported field-dependent Raman data.

Finally, one may argue that vibronic coupling affects ground and excited *g*-factors in a way that may depart from the usual spin-Hamiltonian description of Supplementary Eq. (5). To check this point, we employed the Hamiltonian of Supplementary Eq. (4) and computed the ground- and excited-state KD *g*-factors in dependence of E/D in a field of $B = 0.25$ T in all three directions x, y and z and compared the results with simple calculations using only the usual spin-Hamiltonian of Supplementary Eq. (5). In Supplementary Fig. 3, the results obtained using the latter approximation (solid lines) have been compared with the one accounting for vibronic coupling (black points). For the excited-state KD, deviations are present. But, for E/D smaller than 0.25, they are not significant (Supplementary Fig. 3). Vibronic coupling leads to significantly lower values of g_z and g_y for E/D larger than 0.25. The trend of these changes follows Frank Ham's theorem of vibronic reduction of electronic quantities (the Ham effect)[2-4].

Supplementary Note 4. Additional calculations of spin-phonon coupling constants

The spin-phonon entanglement can be easily rationalized employing a model of the M_S = -1/2 → +3/2 magnetic excitation, $|-1/2,0,0,0\rangle \rightarrow |3/2,0,0,0\rangle$ (both levels in their ground vibrational states) into three singly, excited vibrational states with the same magnetic ground state M_S = -1/2: $|-1/2,0,0,0\rangle \rightarrow |-1/2,1,0,0\rangle, |-1/2,0,1,0\rangle, |-1/2,0,0,1\rangle$. Notice that the magnetic transition M_S = -1/2 → + 3/2 is magnetic-dipole-forbidden, but the three vibrationally excited ones (in the approximation of the harmonic oscillator) are formally allowed. The interaction of these modes with increasing fields requires the use of an effective spin-vibrational Hamiltonian such as that in Supplementary Eqs. (4-6).

Under these assumptions, magnetic and phonon positions, with magnetic fields oriented parallel to the *z*, *x* and *y* canonical axes, have been computed and plotted in Fig. 5 (main text, *z* axis) and Supplementary Figs. 4a (*x* axis) and 4b (*y* axis). Inspection of the plots allows the identification of data points which strictly follow a linear *B* vs E dependence and are subjected to avoided crossings reflecting spin-phonon entanglement. The $B||z$ plot nicely reproduces the three avoided crossing regions with point positions compared with the experimental points in Fig. 5 of the main text.

Field directions parallel to the *x* and *y* axes lead to resonances at higher fields that do not match the experimental pattern.

Lastly, peak A in Raman spectra of **1** and **1-*d*$_4$**, dominated by a phonon shifting to larger energies in high fields (Figs. 4a-b of the main text, black points), has been analyzed using the single-mode model (ref. 22 in the main text). In this model, a phonon at 114 cm^{-1} interacts with the magnetic excitation at 115 cm^{-1} with off-diagonal element E_1 = 1 cm^{-1} which mixes the two wavefunctions at 0 T. Due to this mixing, the vibrational state acquires some magnetic character,

which is responsible for the high-field-high-frequency shift of the resonance. It is invariably observed in the Raman spectra of **1** and **1-*d*₄**. As shown in Supplementary Fig. 5, the single-mode-coupling model nicely reproduces this feature for all three field directions $B||z$, $B||x$ and $B||y$.

Supplementary Note 5. Mechanism of the intensities in the field-dependent Raman spectra

Spin-phonon coupling manifested in the avoided crossings (Fig. 4, main text) and its simulation (Fig. 5, main text) using the vibronic coupling model of Supplementary Eqs. (4-6) raise the question about the underlying vibronic wavefunctions which also affect the intensity of the Raman transitions. These vibronic wavefunctions are linear combinations of the basis functions expressed as products of the magnetic sublevels belonging to the $S = 3/2$ spin and harmonic oscillator wavefunctions as described in the main text, Methods section, Eqs. (7.1-7.17). Transitions from the ground-vibronic state dominated by the contribution of $M_S = -1/2$ into the manifold of vibronic states stemming from the coupling of the $M_S = 3/2$ spin sublevel with the magnetic field $B||z$ are expected to be governed by both Raman- and magnetic-dipole-allowed contributions to the intensity. In Supplementary Fig. 6, we show the field dependence of the intensity for **1**.

As discussed in the text, the magnetic peak is not observed at fields up to 6 T. This is in accordance with the dominating magnetic-dipolar nature of the transitions, which is of lesser intensity than Raman-allowed ones. Starting with $B = 6$ T, the intensity of the data points of branch B (red circles) can be identified as being dominated by the vibrational component of the vibronic transitions of the weakly intense, vibrational-excited state, 125 cm^{-1}. Raman peaks of branch D (green circles) at zero field (128 cm^{-1}) are with a bigger intensity. However, they lose in intensity when the percentage of their magnetic character increases at the higher fields. At the same time, the Raman peaks of branch C (blue circles) gain intensity at higher fields where the corresponding vibronic wavefunction becomes dominated by a singly excited vibrational mode, 128 cm^{-1} belonging to branch D in zero field. Thus, the composition of the vibronic wavefunction changes with the field. This induces a redistribution of intensity between Raman

peaks in branches B, C and D. To simulate this behavior in a rough but correct way, we have made explicit use of the computed wavefunctions where, out of the all possible transitions, only the component of the product of vibronic wavefunctions with contribution from M_S = -1/2,0,0,0 → M_S = -1/2,0,1,0 (128 cm^{-1}, belonging to branch D in zero field) is taken into account, thus approximating the intensities of branches B, C and D using the expression:

$$I_i = \left[c_i(B,-1/2,0,1,0)c_1(B,-1/2,0,0,0)\right]^2 \tag{7}$$

with i = B,C,D and c_1 referring to the ground-vibronic state.

Using this approximation, we qualitatively to semi-quantitatively reproduce Supplementary Fig. 6 using the field dependence of the intensity (Supplementary Fig. 7).

Supplementary Note 6. Analysis of far-IR transmittance spectra

In the transmittance far-IR spectra of powder samples of **1**, **1-*d*$_4$** and **1-*d*$_{18}$** (Supplementary Figs. 8-11), the magnetic features of the coupled peaks (from the couplings between the magnetic transitions and *g* phonons in Fig. 3f in the main text) are qualitatively observed. One of the two coupled peaks, not well resolved, shifts quickly with the field. This is the one that carries most of the magnetic features (i.e., the ZFS transition). The other coupled peak (at the same field) carries mostly the features of a *g* phonon and thus does not shift much with the fields. The magnetic features of the peak show up in far-IR. For example, the 115 cm^{-1} peak of **1-*d*$_4$** in Supplementary Figs. 8c-d and 10 is from the magnetic features of phonon peak A. It only gradually decreases in intensity with field increase, as its magnetic features gradually decrease with field increase. However, it should be pointed that for the powder sample of **1-*d*$_4$**, some powders may orientate along the *x*-direction. The $\phi_1|0\rangle \rightarrow \phi_3|0\rangle$ magnetic transition along the *x*-direction (similar to trace 2 in Fig. 6b of the main text) may overlap with magnetic features of *g* phonon peak A here.

There are field-sensitive *u* phonons in the far-IR spectra of all three isotopologues. In **1** (**1-*d*$_4$**), the two phonons at ~125 (124) and ~134 (134) cm^{-1} near the spin-phonon coupled peaks both increase in energy with field. Such hardenings have been reported in, e.g., ferromagnetic MOF-like $Co[N(CN)_2]_2$ (ref. 47 in the main text). Unlike the *g* phonons that undergo spin-phonon couplings (and discussed in the main text), these hardening *u* phonons do not change the spin of the molecule through their molecular or lattice vibrations. The 125 cm^{-1} phonon is predominantly a scissoring motion of the two H_2O ligands. The second phonon at 134 cm^{-1} is mostly a Co-O(acac) stretching mode where opposite O atoms have an antisymmetric stretching with the cobalt atom, as VASP phonon calculations below in Supplementary Note 7 show. The

other two O atoms do not shift noticeably. The 123 cm^{-1} phonon in **1-*d*$_{18}$** is a similar mode to the 125 cm^{-1} mode in **1** and **1-*d*$_4$**, in which a scissoring motion of the two H_2O ligands occurs. We focus only on the local coordination sphere of the cobalt atom, despite these not being localized vibrations.

Supplementary Note 7. Additional results of the periodic DFT phonon calculations

The very low-energy region is full of intermolecular phonon modes whose frequencies are very sensitive to the accuracy of the crystal structure model and the intermolecular interactions. Frequency calculation of these modes is well known to be challenging. Therefore, it is no surprising that the match between experiment and calculations is not perfect. Due to the centrosymmetric nature, phonon peaks that are observed in Raman spectra will not be observed in far-IR and vice versa. However, we found in our data that if a *g* symmetry mode is coupled to the ZFS peak, it is allowed in far-IR.

The modes and thus atomic displacements of **1** and **1-*d*₄** are similar in the region of the ZFS peak (Supplementary Fig. 12). It is evident from the phonon calculations that there is an A_g/B_g phonon (peak A) near the ZFS peak in the Raman spectra of **1**, **1-d_4** and **1-d_{18}**. The phonon calculations are used to understand the nature of the phonon overlapping with the ZFS peak in the far-IR spectra. The calculated peak position suggests there is no A_u or B_u phonon mode directly overlapping with the ZFS peak (~115 cm^{-1} for **1-d_4** and ~112 cm^{-1} for **1-d_{18}**) in far-IR. However, the phonon calculations of the peak positions show two phonons of *u* symmetry in the 115 cm^{-1} region (i.e., 114.3 and 116.3 cm^{-1}, Supplementary Table 3) for **1-d_{18}**. Experimentally, these features are weak (Supplementary Fig. 8). In order to show the weakness in intensities of these features, the intensities of the far-IR phonon modes in this region were calculated (Supplementary Fig. 13). It is clear there are not quantifiable peak intensities from phonon modes in this region, suggesting that these peaks must be very weak in far-IR. Far-IR intensities were derived using the method by Gianozzi and co-workers[5]. The results here suggest that the intensity gradually disappearing as the ZFS peak shifts away is of *g* symmetry and not formally allowed in far-IR. As the ZFS moves further away, the coupling to this *g* symmetry becomes

weaker and hence disappears as it is not formally allowed.

The methyl hydrogen (deuterium) atoms have the largest displacements out of any atoms in the phonons near the ZFS peak, i.e., A, C, D and E, of **1-d_4** (A and B of **1-d_{18}**). However, due to the distance to from these atoms to the Co^{II} center (~4.5-5 Å), it is hard to imagine they have a large role in magnetic relaxation. Therefore, in the case of the aforementioned phonons, these cannot be used to explain the differences in the coupling constants $|\mathit{\Lambda}|$. Supplementary Table 6 shows the change in the O-Co-O equatorial bond angle compared to $|\mathit{\Lambda}|$ for the relevant phonon modes. It is clear there is a trend in the magnitude of $|\mathit{\Lambda}|$ and the distortion of the O-Co-O equatorial bond angle. These bond angles are determined from the geometry-optimized crystal structure.

Supplementary Note 8. Origin of the orthorhombic splitting in $Co(acac)_2(H_2O)_2$ (1)

The presence of two different oxygen ligators in the coordination sphere of the Co^{II} ion leads to lowering of the symmetry of the six-fold coordinate Co^{II} from O_h to the observed C_{2h} in **1**. The pseudo-tetragonal D_{4h} component of the ligand field stems from the two-axial water ligands with longer Co-O bonds than the four equatorial Co-O bonds by the two acac anionic ligands. In such a coordination and taking Cartesian axis orientation (*x,y,z*) as shown in Supplementary Fig. 14, the d_{xz},d_{yz} pair of molecular orbitals (with e_g symmetry in D_{4h}) is more stabilized than the d_{xy} MO (with b_{2g} symmetry). Anisotropic Co-O π-interactions, with contributions from the out-of-plane water π-orbital (Supplementary Fig. 15) and intrinsic π-anisotropy due to the π-electronic structure of the acac ligand, leads to the splitting of the d_{xz}/d_{yz} orbital pair into b_1 and b_2 MOs (using the C_2 point group notations), as shown in Supplementary Fig. 16. The C_2 symmetry of each Co-acac moiety leads to the 1:1 mixing of the D_{4h} parent orbitals $d_{yz} \pm d_{xz}$ to produce symmetry-adapted MOs of b_2 ($d_{y'z'}$) and b_1 ($d_{x'z'}$), which interact with corresponding ligand linear combinations of the same symmetry $L_\pi(b_2)$ and $L_\pi(b_1)$, respectively. The latter orbitals have different energies leading to different perturbations of the b_2 ($d_{y'z'}$) and b_1 ($d_{x'z'}$) metal-based orbitals as Supplementary Fig. 16 shows. This leads to a difference in the antibonding energy of $d_{y'z'}$ (e_π) and $d_{x'z'}$ (e'_π). Perturbation theory to second order yields the following expressions for e_π and e'_π:

$$e_\pi = \frac{\langle 3d_{y'z'}(b_2)|h|L_\pi(b_2)\rangle^2}{E(d_{y'z'}) - E(L_\pi(b_2))} \quad (8)$$

$$e'_\pi = \frac{\langle 3d_{x'z'}(b_2)|h|L_\pi(b_1)\rangle^2}{E(d_{x'z'}) - E(L_\pi(b_1))} \quad (9)$$

As has been shown elsewhere[6,7], $E(d_{y'z'}) - E(L_\pi(b_2)) < E(d_{x'z'}) - E(L_\pi(b_1))$, and $\langle 3d_{y'z'}(b_2)|h|L_\pi(b_2)\rangle \cong \langle 3d_{x'z'}(b_2)|h|L_\pi(b_1)\rangle$ leading to $e_\pi > e_\pi^{'}$ (Supplementary Fig. 16). For $Co(acac)_2(H_2O)_2$ (**1**), because of the presence of two acac ligands, the splitting of the $d_{y'z'}$,$d_{x'z'}$ orbital set is given by twice the energy difference $e_\pi - e_\pi^{'}$. Interestingly enough, the orientation of the two water molecules in the Co^{II} coordination sphere adds a positive contribution to this splitting. As shown in Supplementary Fig. 15, the two water ligands are aligned with their π-lone pairs almost parallel to metal-based $d_{y'z'}$ orbital which is destabilized with the respect to the $d_{x'z'}$ MO. For latter orbital, there are no orbitals within the plane of the H_2O ligands available for π-interactions with the Co^{II} ion. An *ab initio* ligand-field diagram, accounting for Co-ligand interactions of both σ- and π-types (Supplementary Fig. 17), shows the C_{2h} low-symmetry splitting of the 3d-based MOs of the Co^{II} ion. When extended to a many-electron description of the d^7 Co^{II} ion, these one-electron effects lead splitting of the octahedral $^4T_1$ ground state of octahedral Co^{II} into three non-relativistic sublevels at 0 (^4A_g), 707 cm^{-1} (^4B_g) and 1175 cm^{-1} (^4B_g) (Supplementary Table 7). When accounting for spin-orbit coupling, this results in D = 89.4 cm^{-1} and large E/D = 0.17 parameters. See also Supplementary Table 8.

Supplementary references

1 TOPAS-Academic V6 (http://www.topas-academic.net/ accessed on October 8, 2017) (2016).

2 Ham, F. S. Dynamical Jahn-Teller effect in paramagnetic resonance spectra: Orbital reduction factors and partial quenching of spin-orbit interaction. *Phys. Rev.* **138**, A1727-A1740 (1965).

3 Ham, F. S. in *Electron Paramagnetic Resonance* (ed Geschwind, S.) Ch. 1, 1-119 (Plenum Press, New York and London, 1972).

4 Bersuker, I. *The Jahn-Teller Effect and Vibronic Interactions in Modern Chemistry*. (Plenum Press, New York and London, 1984).

5 Giannozzi, P. & Baroni, S. Vibrational and dielectric properties of C_{60} from density-functional perturbation theory. *J. Chem. Phys.* **100**, 8537-8539 (1994).

6 Ceulemans, A., Dendooven, M. & Vanquickenborne, L. G. The ligand field of phase-coupled ligators. *Inorg. Chem.* **24**, 1153-1158 (1985).

7 Atanasov, M. A., Schönherr, T. & Schmidtke, H.-H. The role of the π-bonding network for trigonal level splittings of tris-bidentate $Cr(acac)_3$ and $Cr(ox)_3^{3-}$. *Theor. Chim. Acta* **71**, 59-73 (1987).
