# Supplementary material for: Spin–phonon couplings in transition metal complexes with slow magnetic relaxation
Source: Nat Commun. 2018 Jul 3;9:2572. doi: 10.1038/s41467-018-04896-0 (PMC6030095; doi:10.1038/s41467-018-04896-0)
Supplement: Supplementary file 2 — Description of Additional Supplementary Files [file 41467_2018_4896_MOESM2_ESM.pdf]

Description of Additional Supplementary Files

File Name: Supplementary Movie 1
Description: Movie 1 of phonon A in **1-*d*$_4$** at 109.2 cm^{-1}

File Name: Supplementary Movie 2
Description: Movie 2 of phonon C in **1-*d*$_4$** at 126.0 cm^{-1}

File Name: Supplementary Movie 3
Description: Movie 3 of phonon D in **1-*d*$_4$** at 129.3 cm^{-1}

File Name: Supplementary Movie 4
Description: Movie 4 of phonon E in **1-*d*$_4$** at 142.7 cm^{-1}

File Name: Supplementary Movie 5
Description: Movie 5 of phonon A in **1-d$_{18}$** at 116.3 cm^{-1}
