# Supplementary figures and images for: Spin–phonon couplings in transition metal complexes with slow magnetic relaxation
Source: Nat Commun. 2018 Jul 3;9:2572. doi: 10.1038/s41467-018-04896-0 (PMC6030095; doi:10.1038/s41467-018-04896-0)

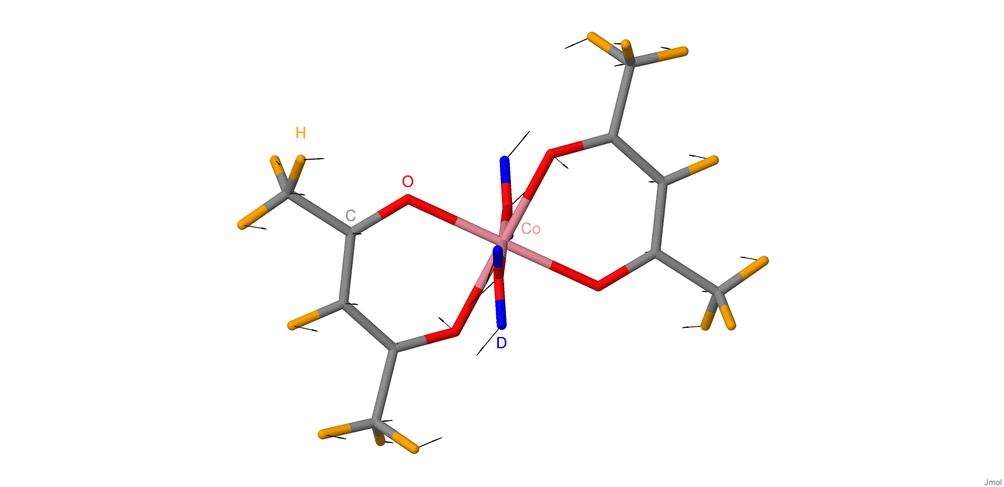

Supplement: Supplementary file 3 — Supplementary Movie 1 [file 41467_2018_4896_MOESM3_ESM.gif]

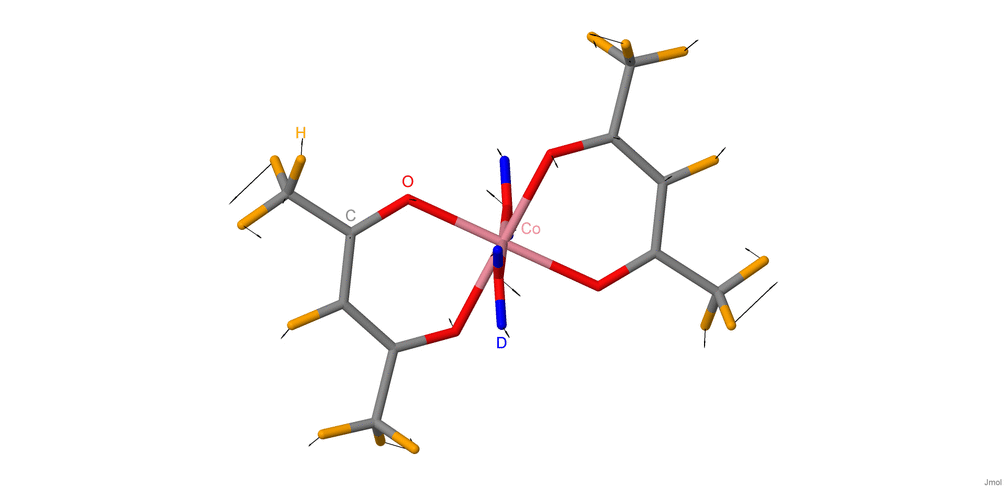

Supplement: Supplementary file 4 — Supplementary Movie 2 [file 41467_2018_4896_MOESM4_ESM.gif]

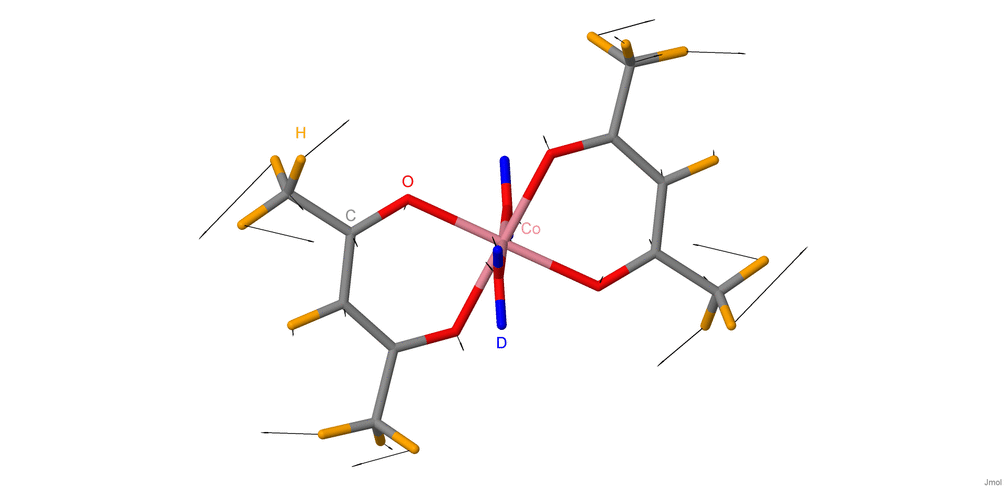

Supplement: Supplementary file 5 — Supplementary Movie 3 [file 41467_2018_4896_MOESM5_ESM.gif]

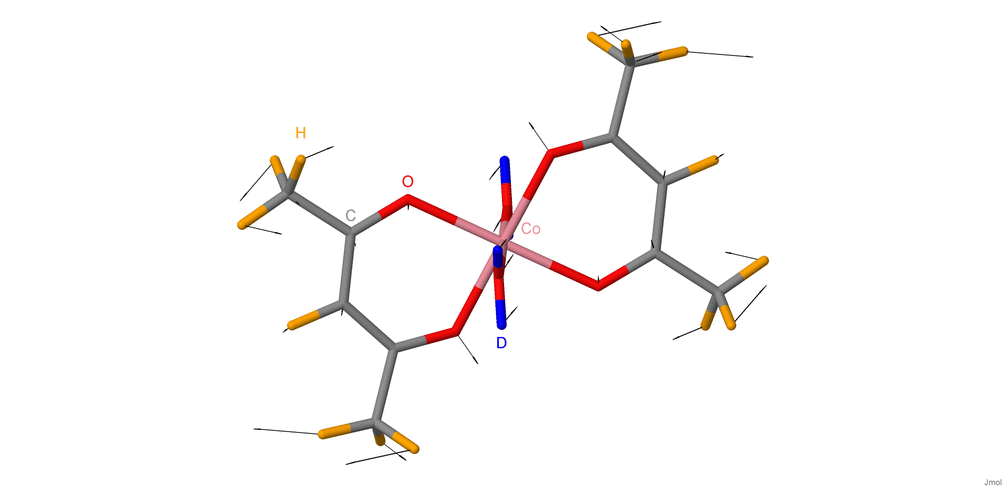

Supplement: Supplementary file 6 — Supplementary Movie 4 [file 41467_2018_4896_MOESM6_ESM.gif]

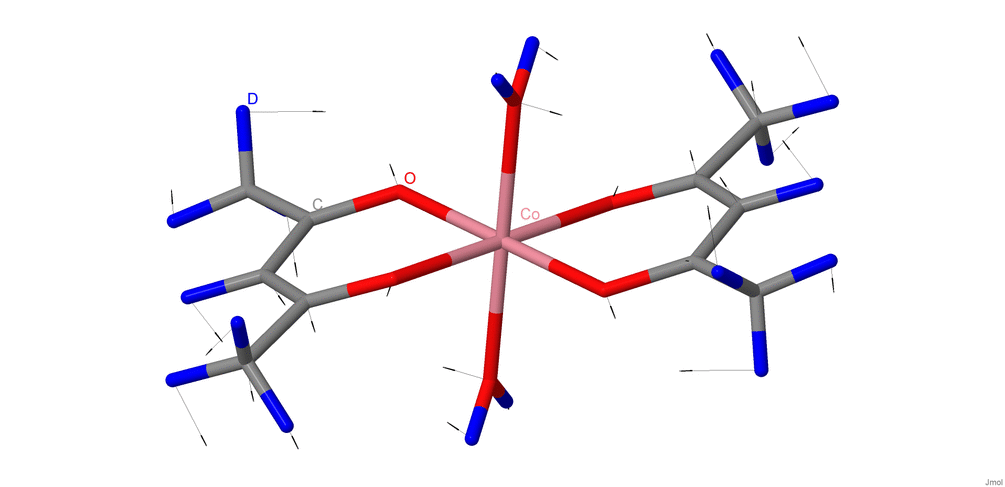

Supplement: Supplementary file 7 — Supplementary Movie 5 [file 41467_2018_4896_MOESM7_ESM.gif]
